# Personalized signaling pathway analysis of gastrointestinal tumors for patient stratification and drug target evaluation using clinically derived core biopsies

**DOI:** 10.1038/s41698-026-01304-5

**Published:** 2026-02-25

**Authors:** Aaron Stahl, Karsten Büringer, Pavlos Missios, Tatjana Hoffmann, Sven Mattern, Stephan Singer, Felix Schäfer-Ruoff, Nisar P. Malek, Katja Schenke-Layland, Michael Bitzer, Markus F. Templin

**Affiliations:** 1 NMI Natural and Medical Sciences Institute at the University of Tübingen, Reutlingen, Germany; 2 Institute of Biomedical Engineering, Department for Medical Technologies and Regenerative Medicine, University of Tübingen, Tübingen, Germany; 3 Department of Internal Medicine I, University Hospital Tübingen, Tübingen, Germany; 4 Center for Personalized Medicine, Eberhard-Karls University, Tübingen, Germany; 5 Department of Pathology and Neuropathology, University Hospital Tübingen, Tübingen, Germany; 6 M3-Research Center for Malignome, Metabolome and Microbiome, Eberhard-Karls University, Tübingen, Germany

**Keywords:** Biomarkers, Cancer, Computational biology and bioinformatics, Gastroenterology, Oncology

## Abstract

Aberrant cellular signaling underlies cancer development and progression. Identifying alterations in these pathways yields critical insights for personalized oncology. Clinically, assessing the activation status of signaling proteins complements genetic and histopathological analyses, improving therapeutic evaluation and accuracy. In this study, we employed the high-throughput Western blot system DigiWest for the characterization of gastrointestinal tumors, both retrospectively and in a proof-of-concept direct clinical application. Retrospective analyses of pancreatic and colorectal carcinomas (*n* = 20) compared with matched normal tissues revealed distinct protein expression and activation patterns differentiating tumor subtypes and defining clinically relevant subgroups. By resolving individualized, treatment-relevant signaling signatures we demonstrate the feasibility of molecular-level personalization in samples with high clinical heterogeneity. In the clinical proof-of-concept, single core needle biopsies from 14 patients with gastrointestinal tumors who underwent Molecular Tumor Board presentation were analyzed. The resulting proteomic profiles uncovered patient-specific, targetable pathway activation patterns and showed concordance with mutational data and therapy recommendations. Collectively, these findings establish DigiWest as a valuable, robust complementary tool to sequencing-based approaches for personalized diagnostics and treatment evaluation in precision oncology.

## Introduction

Our mechanistic understanding of cancer is heavily reliant on the identification of aberrantly regulated signaling pathways, as tumor-acquired genetic aberrations (e.g. point mutations, deletions, amplifications, gene fusions or CNVs) manifest as changes in pathway activity on the protein level. In light of this, signal-transducing proteins have become a primary target for therapeutic intervention^1^. Especially in the age of personalized medicine, the identification of pathway-based activity patterns can not only serve as a basis for patient stratification within and across various tumor entities but also opens opportunities for individualized tumor characterization and personalized treatment evaluation. In recent decades, a large variety of drugs have been developed targeting various pathways such as MAPK^2^, PI3K/Akt/mTOR^3,4^, or cell cycle regulation^5^ as well as upstream tyrosine kinase receptors including EGFR^6^, FGFR^7,8^, VEGFR^9^ or Her2^10^. With regulatory approval of such drugs (as mono- or combination therapies) along with immunotherapeutic approaches^11–13^ for use across tumor entities^14^, clinical oncologists now have a vast array of treatment options available, including the potential of off-label therapies. At the same time, significant progress in sequencing technologies and genome profiling^15,16^ has made genetic mutation analyses a key tool for translational oncology programs (e.g. molecular tumor boards). However, with increasing complexity in genome profiling, clinical interpretation and thus identification of potential tumor drivers has also become more challenging^17^. Selecting the most suitable therapeutic option in each individual case is crucial, especially for patients with recurring tumor or diagnoses at advanced disease stages. In recent years, the transfer of personalized approaches into clinical application have mainly focused on genetic mutation analyses. However, pathway activity changes ultimately manifest on the proteome level rendering the implementation of personalized proteomic approaches especially crucial. To do so—and given the complexity of signaling networks – insight into protein expression and posttranslational modification (e.g. phosphorylation, methylation, acetylation, cleavage) is required at a large scale. Many protein analytics methods such as standard and multiplexed immunohistochemistry^18,19^, Reverse-Phase Protein Arrays (RPPA)^20^ and Mass Spectrometry either lack the throughput required for extensive signaling pathway analysis and/or demand high sample amounts^21^. These crucial drawbacks leave proteomic methods still heavily underrepresented in clinical practice. Thus, proteomic analysis of molecular drug targets and cellular signaling entails a promising approach for individualized tumor profiling and subsequent treatment evaluation. Gastrointestinal (GI) cancers encompass some of the most common and lethal forms of cancer including pancreatic and colorectal carcinomas. Alike other GI tumors (e.g. esophagus, liver, stomach, gallbladder) they are often diagnosed at advances stages and treatment options remain limited^22^, thus leaving an urgent need for novel, personalized therapy approaches. Commonly altered signaling pathways in pancreatic, colorectal and other GI cancers for instance include MAPK/Erk, PI3K/mTOR/Akt, TGF beta/Smad, Wnt/beta-catenin, Notch or Jak/STAT signaling^23,24^. At the genome level, most pancreatic carcinomas ( > 90%) harbor mutations in the KRAS oncogene^25^; other commonly mutated or deleted genes are the tumor-suppressors TP53, SMAD4 and CDKN2A^26^ as well as various tyrosine kinase receptors^27^. Common mutations in colon tumors include EGFR, KRAS, PIK3CA, PTEN and TGFBR1/2^28^. Loss of the Wnt regulator APC is also observed in 80% of cases^29^. Despite several of these genetic traits being shared among tumors of the same tissue, both pancreatic and colorectal cancers (as well as other GI tumors) are highly heterogenous. This substantial inter-tumor and inter-patient variability brings about wide-spread tumor behaviors and characteristics affecting therapy response, treatment resistance and patient outcome^22,30^, thus making personalized medicine approaches especially suitable for this class of malignancies. Given the complexity of signaling networks and the fact that their activation status distinguishes true from potential tumor drivers, this calls for the integration of intracellular signal transduction analysis as it may define individualized treatment options and stratify patients beyond the capabilities of genetic and transcriptomic analysis. The DigiWest is a high-throughput Western Blot variation, which allows concomitant detection of up to 200 proteins and phosphorylated protein variants from minimal amount of sample while retaining the sensitivity of classical Western Blotting^31^. It has previously been employed for signal transduction analysis of both cellular in vitro cancer models and primary tumor tissue^32–36^, for instance for the expression-based stratification of mammary carcinomas based on immune-cell-related protein signatures^37^. Here, we have developed an antibody panel tailored to the analysis of gastrointestinal tumors encompassing > 130 proteins and phospho-proteins for extensive signaling pathway analysis. In a first step, we use this panel consisting of major drug targets, key signaling proteins, tyrosine kinase receptors, tumor markers, and immune cell markers to retrospectively characterize archived primary tumor tissue from pancreatic and colorectal carcinomas. Using patient-specific, normal tissue-matched expression data, we conceptually show the suitability of the panel and methodology by stratifying these tumors based on cellular signaling and clinical data. We also create individualized protein profiles for each tumor. Secondly, in an exploratory direct clinical application based on patient-derived tissue core biopsies, we transfer this personalized proteomics approach to a prospective case series of molecular tumor board cases highlighting the potential of DigiWest to suggest individual treatment options.

## Results

### Absolute and relative expression differences between pancreatic and colorectal tumor tissues

**Fig. 1:**
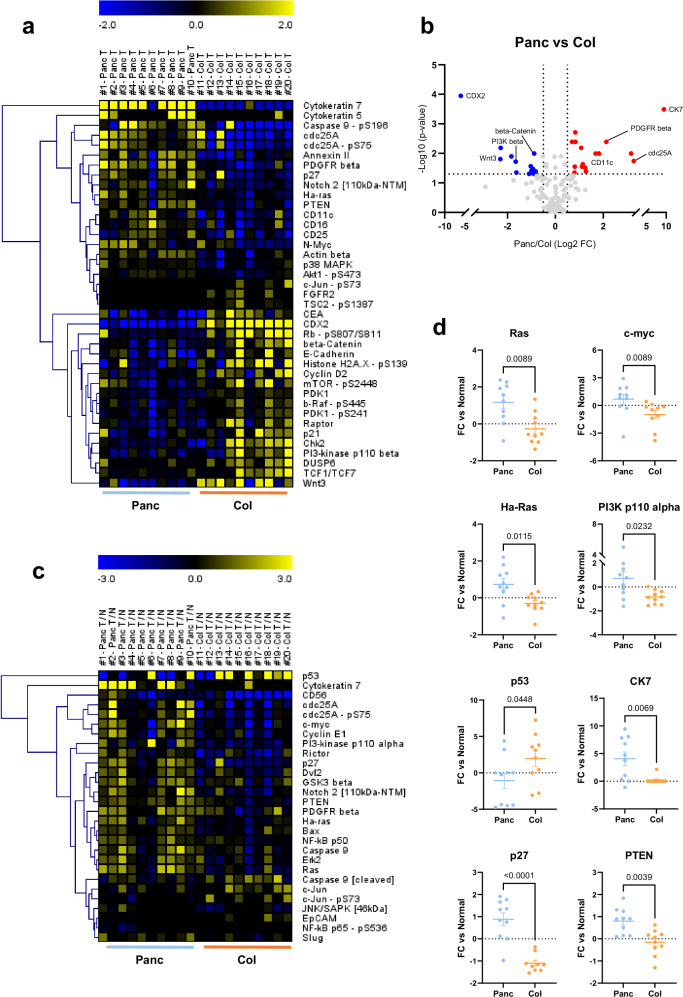
Comparison of pancreas and colorectal carcinomas. **a** Heatmap and Hierarchical Cluster analysis of analytes significantly different between pancreas (*n* = 10) and colon (*n* = 10) tumor tissues using AFI (accumulated fluorescent intensity) signals; Wilcoxon test, *p* < 0.05. **b** Volcano plot of comparison shown in A. Significantly upregulated proteins are shown in red, downregulated proteins in blue. Analytes with FCs < I0.5I are excluded. **c** Heatmap and Hierarchical Cluster analysis of analytes significantly different between pancreas (*n* = 10) and colon (*n* = 10) samples based on expression changes relative to matched normal tissue (as Log2 FC tumor/normal); Wilcoxon test, *p* < 0.05. **d** Tumor/normal relative DigiWest data (Log2 FCs) for selected differentially expressed analytes; Mann-Whitney test, *p* < 0.05. Panc = Pancreas (blue), Col = Colon (orange). *p* value as indicated. Solid line indicates the mean FC value per group. Error bars: S.E.M.

In the first part of our study, we use our developed antibody panel to characterize the retrospective sample cohort in detail (for complete analysis see Supplementary Figure S1). As an initial analysis, pancreatic (*n* = 10) and colorectal (*n* = 10) tumors were compared using absolute DigiWest expression data, thus without factoring in normal tissue. Hierarchical cluster (HCL) analysis indicated an almost ideal separation of tumor samples according to their origin tissue (Supplementary Figure S2a). Of the 137 measured analytes, 39 (28.4%) were differentially expressed between the two tumor types, (Fig. 1a). Expectedly, we observed high, consistent expression differences for tissue markers such as Cytokeratin 7 (pancreas) and CDX2 (colon, Fig. 1b, Supplementary Figure S2b-c). Pancreas carcinomas generally showed greater expression of cell cycle-regulating proteins, immune cell markers or PDGFR beta while expression of Wnt and mTOR-signaling proteins was higher in colon carcinomas (Fig. 1a-b, Supplementary Figure S2b-c). However, when using expression data from tumors only, it is difficult to distinguish between tissue-specific markers and expression differences inherent to the tumors themselves. Next, we compared all tumor tissues (*n* = 20) to their patient-matched normal tissue (*n* = 20, Supplementary Figure S3a). Here, we observed consistently elevated expression of the tumor marker cancer embryonic antigen (CEA) and several other tumor-associated proteins (e.g. BcL-xL, CDK2, beta-Actin, FN1) for both tumor types (Supplementary Figure S3b).

Based on this, all data from tumor tissues were now solely regarded in relation to its respective normal tissue (as Log2 Foldchange). Thus, each individual datapoint indicates a relative expression change occurring from non-cancerous to cancerous tissue stemming from the same patient. When comparing the two tumor entities in this fashion, 28 analytes (20.4%) were significantly different between them (Fig. 1c), only 7 of which were also found when comparing tumor tissues directly (see Fig. 1a). Furthermore, tumors no longer clustered according to their tissue origin (Supplementary Figure S4) and tissue-specific analytes such as CDX2 now expectedly showed no differential expression (Supplementary Figure S5a). Crucially, the pancreas carcinomas on average showed upregulations of several proto-oncogenic proteins such as Ras (KRAS), c-myc (MYC), Ha-Ras (HRAS) or PI3K alpha (PIK3CA), among others (Fig. 1d and Supplementary Figure S5b). Moreover, strong downregulation (5-fold) of the tumor suppressor protein p53 (TP53) was observed to a greater extent (Fig. 1d). Lastly, CK7 was drastically upregulated (up to 10-fold) in some pancreatic tumors. On the other hand, colon carcinomas for instance displayed strongly reduced levels of the tumor suppressors p27 (CDKN1B) and PTEN (Fig. 1d). However, substantial variability within the cohorts is generally worth noting (e.g. see p53, Ras). Thus, when including normal tissue as a reference, the tumor entities could be distinguished largely based on protein expression changes of key tumor suppressors and oncogenes, placing emphasis on a pathway-activity based distinction. Next—again using relative data—we stratified tumors within their respective cohorts.

### Relative expression changes distinguish samples within the pancreas carcinoma cohort

**Fig. 2:**
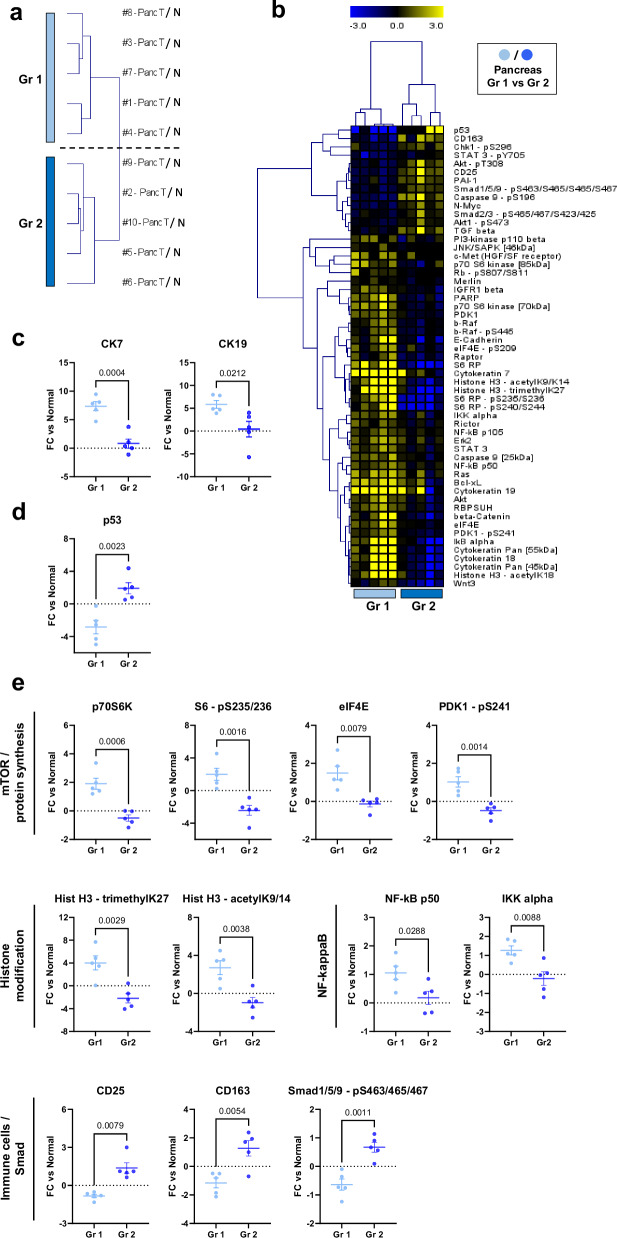
Relative expression differences within the pancreas cohort. **a** Hierarchical Cluster analysis of pancreas tumors only (*n* = 10) using relative expression data (Log2 FC tumor/matched normal); Group 1 = Gr1 (light blue), Group 2 = Gr2 (dark blue). **b** Heatmap and Hierarchical Cluster analysis of analytes significantly different between Gr1 (*n* = 5) and Gr2 (*n* = 5) pancreas tumors; Wilcoxon test, *p* < 0.05. **c**–**e** Tumor/normal relative DigiWest data (Log2 FCs) for **c:** Cytokeratins 7 and 19, **d** p53, and **e** selected analytes of interest with their pathway allocation; either the Mann-Whitney test or unpaired t-test was used depending on data distribution. p-value as indicated. Solid line indicates the mean FC value per group. Error bars: S.E.M.

Cluster analysis clearly separated the 10 pancreatic tumors into two subsets of five (Fig. 2a), indicating differential pathway activity in the two groups. Our protein data revealed that tumor-induced changes to a striking 40.1% (55/137) of analytes were significantly different between the two subgroups (Fig. 2b), with 42 of those showing higher changes in magnitude for group/cluster 1 (light blue) and only 13 for group/cluster 2 (dark blue). Notably, group 1 generally showed strong upregulation (ca. 5-fold) of Cytokeratins (Fig. 2c, Supplementary Figure S6a), whereas CK levels were not changed in group 2. Downregulation of p53 was another feature of group 1 carcinomas (Fig. 2d). In addition, group 1 showed an upregulation of several mTOR-pathway-proteins, its downstream targets (Fig. 2e) and several others (Supplementary Figure S6b). They also showed highly increased amounts of modified Histone H3 (Fig. 2e) as well as strong up-regulatory effects on NF-kappaB signaling (Fig. 2e and Supplementary Figure S6c). On average, expression levels of all these analytes were downregulated or unchanged compared to normal tissue in group 2. Conversely, these pancreatic tumors were characterized by higher signals for immune cell markers (Fig. 2e) along with key Smad signaling proteins (Fig. 2e and Supplementary Figure S6d). The subgroups could not be associated with clinical parameters such as TNM stage or patient age. However, it was notable that all poorly differentiated tumors (G3) were part of group 2, whereas group 1 only included moderately differentiated (G2) tumors (Table 1). Overall, upon comparison to normal tissue, pancreatic carcinomas showed a clear separation into two distinct groups based on signaling profiles.

**Table 1:** Summary of clinical and DigiWest data of retrospective patient cohort

Sample		Clinical Characteristics							DigiWest Analysis					Pathology
#	Origin	Patient Age (y)	Pre-Therapy	Histological Differentiation	TNM	Localization	MSI Status	Treatment End to Resection (d)	Markers	Key Pathways	Receptors	Others	Pathway Identified?	Relative Tumor Cell Content (%)
1	Pancreas	74	none	G2	pT1c. pN2 (4/15), Lo, V0, Pn1, R0	pancreas head	n/a	---	CEA+++ CK19+++ CK7+++	p53 loss/cell cycle++ Ras/MAPK/Erk++	c-Met VEGFR Her2	mTOR-p70S6K-S6	Yes	40
3	Pancreas	72	FOLFIRINOX	G2	ypT3, ypN1 (6/31), L0, V1, Pn1, R1	pancreas head	n/a	72	CEA+++ CK19+++ CK7+++	Akt/mTOR++ p53 loss/cell cycle++ beta-catenin++	EGFR Her2 c-Met	histone modifications NF-kB	Yes	60
4	Pancreas	73	none	G2	pT3, pN1 (2/33), L0, V0, Pn1, R1	pancreas head	n/a	---	CEA+++ CK19+++ CK7++ AXII-		c-Met	NF-kB histone modifications protein synthesis+	No	30
7	Pancreas	69	none	G2	pT3, pN1 (1/27), L0, V0, Pn1, R1	pancreas head	n/a	---	CEA+++ CK19+++ CK7+++	Ras/MAPK/Erk/p38++ Wnt/beta-catenin+	EGFR PDGFR	p53 loss histone modifications	Yes	5
8	Pancreas	74	none	G2	pT2, pN1 (1/17), L0, V0, Pn1, R0	pancreas head	n/a	---	CEA+ CK19++ CK7+++ CK5+++	Akt/mTOR++	Her2 EGFR	histone modifications p53 loss/cell cycle NF-kB	Yes	35
2	Pancreas	67	none	G3	pT1c, pN1 (1/17), L0, V0, Pn1, R0	pancreas head	n/a	---	CEA+ CK19++ CK7+++	immune cells++ cell cycle+		NF-kB	Yes	5
5	Pancreas	50	none	G3	pT3, pN2 (8/26), L1, V1, Pn1, R1	pancreas head	n/a	---	CEA+++ CK19+			TGF beta/Smad+ Akt - Caspase 9	No	20
6	Pancreas	84	none	G2	pT3, pN0 (0/10), L0, V0, Pn0, Ro	pancreas head	n/a	---	CEA+++ CK19-- pan-CK--	immune cells++		TGF beta/Smad+ p53++ PIK3CA++	Yes	1
9	Pancreas	59	FOLFIRINOX / FOLFIRI	G2	ypT1c, ypN0 (0/12), L0, V0, Pn1, R0	pancreas head	n/a	55	CEA+++ CK19+++ CK7+	Ras/MAPK/Erk+++ immune cells++	PDGFR	PI3K/Akt/mTOR TGF beta/Smad EMT	Yes	10
10	Pancreas	55	none	G3	pT2, pN2 (8/42), L1, V0, Pn1, R0	pancreas head	n/a	---	CEA- CK7-	immune cells+		EMT p53++	(No)	< 1
11	Colon	51	5-FU + radiation	G2	ypT2, ypN0 (0/15), pMx, L0, V0, R0	left colon	stable	26	CEA+++ CK19+++ CDX2++	EMT		histone modifications immune cells+	Yes	< 1
12	Colon	55	none	G2	pT3, pN0 (0/26), L0, V0, R0	right colon	stable	---	CEA++	immune cells+		TGF beta/Smad+ Akt - Caspase 9	Yes	75
13	Colon	72	FOLFIRI	G3	pT3 (m), pN1 (1/16), pM1 (omentum), L1, V0, R0	left colon	stable	24	CEA+++	MAPK/Erk+++ Jak/STAT+++	EGFR PDGFR		Yes	20
14	Colon	86	none	G2	pT4a (serosal breakthrough), pN0 (0/16), L0, V0, R0	left colon	stable	---	CEA+++ CDX2++	Akt/mTOR ++	c-Met	TGF beta/Smad+ p53+++	Yes	90
15	Colon	77	none	G2	pT3, pN0 (0/18), L0, V0, R0	right colon	stable	---	CEA++	cell cycle++	Her2		Yes	50
16	Colon	30	none	G2	pT2, pN0 (0/24), L0, V0, R0	right colon	high	---	CEA+++ CK7++			mostly downregulations Caspase 9 immune cells	No	80
17	Colon	77	radiation	G2	ypT3, ypN0 (0/14), L0, V0, R0.	right colon	stable	4	CEA+++			mostly downregulations Caspase 9	No	15
18	Colon	26	cisplatin / etoposide / ifosfamide	G3	pT2, pN1b (2/26), pM1 (liver), V1, L0, R0 (local)	right colon	stable	59	CEA+++ CK19+++ CDX2+++ AXII++	Ras/MAPK/Erk/p38++ Akt/mTOR++	Her2	histone modifications Wnt/beta-catenin+ select immune cells	Yes	10
19	Colon	78	none	G2	pT3, pN0 (0/15), L0, V0, Pn0, R0	right colon	stable	---	CEA+++	developmental pathways+		select immune cells	Yes	50
20	Colon	72	none	G2	pT2, pN1 (1/12 LK), pMX, L0, V0, R0	left colon	stable	---	CEA+++	Akt/mTOR++ Wnt/beta-catenin++		cell cycle++ p53+++	Yes	80

For each patient (*n* = 20), relevant available clinical data is shown (left) as well as relevant marker proteins, upregulated key pathways and receptors (right). Yes = coherent pathway activity, No = no clear indication of pathway activity, (No) = sample with inconclusive marker pattern (CEA downregulation). Identifications are based on DigiWest data in relation to respective normal tissue (as Log 2 FC). Most apparent or unique expression signatures/sample are indicated in bold. Far right: Tumor cell content (retrospectively assessed). Blue shadings for pancreatic carcinomas are representative of sub-grouping as identified in Fig. 2. + = relative upregulation versus matched normal tissue, - = relative downregulation.

### Clinical data can be linked to relative expression changes within the colorectal carcinoma cohort

**Fig. 3:**
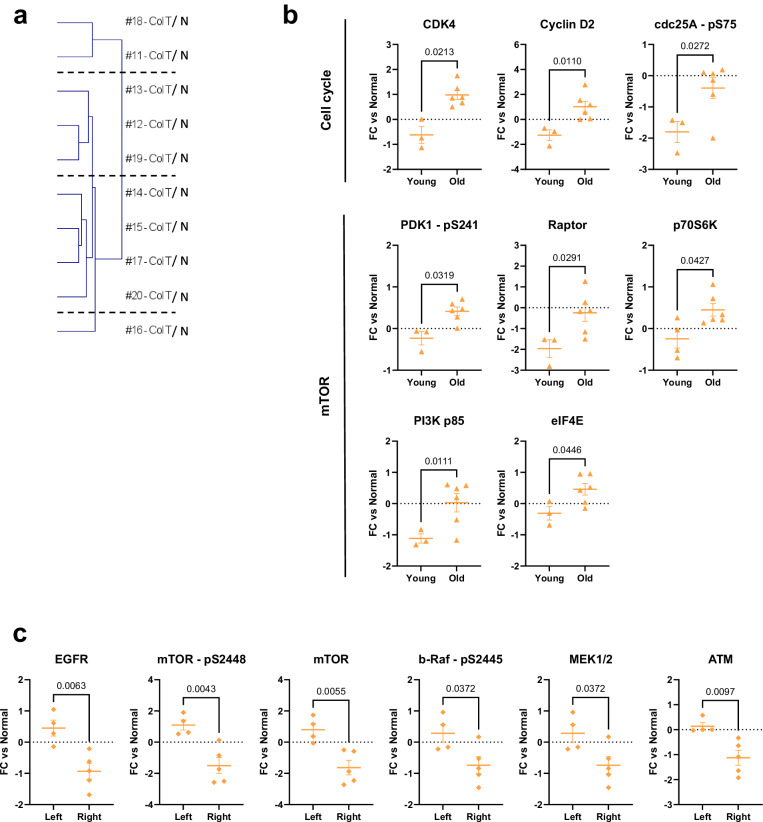
Relative expression differences within the colon cohort. **a** Hierarchical Cluster analysis of pancreas tumors only (n = 10) using relative expression data (Log2 FC tumor/matched normal). **b**, **c** Tumor/normal relative DigiWest data (Log2 FCs) for differentially expressed analytes comparing groups of tumors based on clinical characteristics. **b** Patient age - young ( < 55 years, *n* = 3) versus old ( > 55 years, *n* = 6). **c** Tumor localization - left (*n* = 4) versus right (*n* = 5). Due to differences in group size, Welch´s t-test (*p* < 0.05) was used instead of unpaired t-test. p-value as indicated. Solid line indicates the mean FC value per group. Error bars: S.E.M.

In the colorectal carcinoma cohort, there was more variation with regards to patient age (Supplementary Figure S7a), tumor localization (left vs right sided) and previous treatment, given the available clinical data (Table 1). This comparatively high level of heterogeneity was also evident in the protein expression data, as the ten colon tumors separated into four subgroups upon clustering (Fig. 3a and Supplementary Figure S7b). Therefore, we aimed at stratifying and comparing samples based on patient age and tumor localization, rather than on signaling alone. Due to its unusual clinical nature, we excluded the hepatoid adenocarcinoma sample (#18). Notably, we observed that tumors of young and old patients differed significantly regarding their expression change of cell-cycle-and mTOR-regulating proteins (Fig. 3b). In younger patients ( < 55 y, *n* = 3), their expression was downregulated, whereas upregulations or no changes compared to normal tissue were observed in older patients (*n* = 6). Likewise, right-sided colon tumors (*n* = 5) generally showed downregulations of EGFR, mTOR, MAPK proteins (MEK1/2 and phospho-b-Raf) as well as ATM (Fig. 3c). On the other hand, an upregulation of expression was detected in left-sided (*n* = 4) tumors for these analytes. Previous treatment did not notably affect expression patterns with only 3 analytes showing unpronounced differences (Supplementary Figure 7c). Overall, we were able to show differences in the clinically relevant subgroups of right- and left-sided cancers as well as in the subgroups of early-onset or late-onset colorectal cancers.

### DigiWest highlights distinct expression and pathway activity profiles in individual tumors

**Fig. 4:**
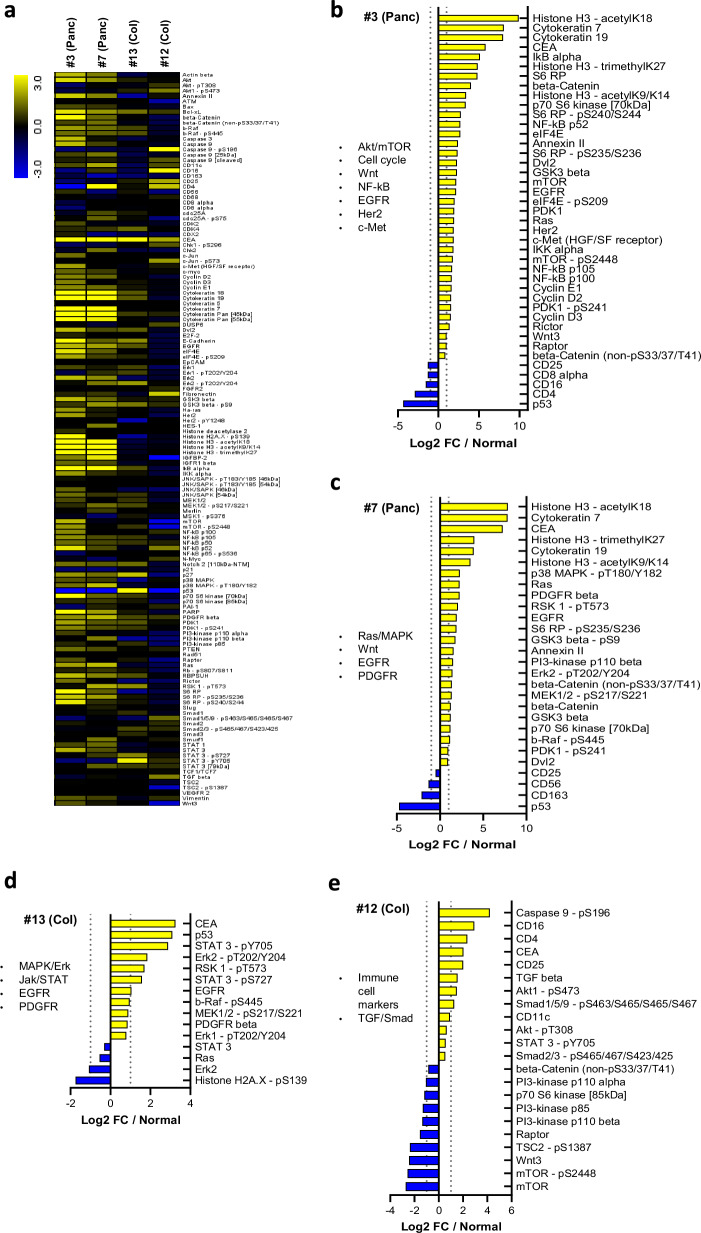
Individual pancreas and colon tumor profiles (exemplary). **a** Heatmap showing tumor/normal relative DigiWest data (Log2 FCs) of all analytes for given example cases (#3, #7, #13, #12). **b-e:** Selection of key up- or downregulated analytes versus matched normal tissue for respective example case. Selection was based on markers and key regulatory (pathway) proteins. Individual bulleted lists indicate affected pathways including potential drug targets. Analogue profiles of all other 16 patients are shown in Supplementary Figs. S8–S23.

Next, we investigated each tumor individually by creating personalized profiles according to dysregulated analytes and analyte groups that infer abnormal signaling pathway activity or impaired cell function. In 15/20 (75%) of cases, based on DigiWest data alone, we were able to assign one or several key pathways and/or tyrosine kinase receptors which could be (substantially) contributing to tumor progression (Table 1). Crucially, of all measured analytes, 37 are direct FDA-approved drug targets (https://www.proteinatlas.org/search/protein_class:FDA+approved+drug+targets), with a further 47 indirectly indicating a target response (e.g. Erk for MEK inhibition). Four exemplary cases are shown in detail in Fig. 4. Analogue protein profiles of all other tumors are shown in Supplementary Figure S8–S23. For each individual tumor, a list of relevant analytes/pathways including potential drug targets is given.

Individual analysis of pancreas carcinoma #3 revealed a variety of expression changes (Fig. 4a). Among the strongest were upregulations of the tumor marker CEA as well as the markers CK7 and 19 (Fig. 4b). Upregulations of several mTOR signaling proteins (e.g. mTOR, PDK1, Rictor), as well as its downstream targets p70S6K, S6 RP and eIF4E (including phospho-variants), indicated substantial activity in this pathway (Fig. 4b). Increased Histone modification, EGFR, Her2 and c-Met expression as well as elevated Wnt (beta-catenin, Dvl2, GSK3 beta, Wnt3) and NF-kappaB signaling (IkappaB, NF-kappaB, IKK alpha) were also noted. Moreover, a strong downregulation ( > 4-fold) of p53 along with upregulated Cyclins E1, D2 and D3 levels drew attention to potentially impaired cell cycle regulation. Pancreas carcinoma #7 exhibited a similar marker expression pattern and displayed coherent activation (phosphorylation) of key MAPK proteins (p38, RSK1, Erk2, MEK1/2, b-Raf), along with elevated Ras, EGFR and PDGFR (Fig. 4a**+c**). In similar fashion, colon carcinoma #13 showed increased CEA, EGFR and PDGFR levels along with upregulated MAPK/Erk and STAT3 phosphorylation (Fig. 4a**+d**). Interestingly, Ras expression was slightly reduced in this case. We were also able to clearly differentiate the only MSI-high tumor (#16) and the hepatoid carcinoma sample (#18,) from the others based on marker expression; they were either the only colon tumor with CK7 upregulation (Supplementary Figure S19) or the one with the highest increase in CK19 (11-fold) and CDX2 (6-fold) (Supplementary Figure S21), respectively.

Finally, we opted to investigate immune cell markers (CDs, for details see Supplementary Table 1) as their expression is often indicative of immune cell infiltration into the tumor (so called “hot” versus “cold” tumors). Across all 20 tumors, groups of CD proteins were consistently upregulated in nine cases (Table 1) in some of which their higher expression being among the most prevalent key changes, potentially indicating an immunologically “hot” tumor. One such example (colon #12) is shown in Fig. 4a**+e**. Notably, CD16, CD4, CD25 and CD11c expression is elevated along with TGF beta, phospho-Smad1/5 and phospho-Smad 2/3, which can play a role in immunogenic signaling. In contrast, expression of proteins from other common signaling pathways (e.g. mTOR, Wnt) was reduced. Immune infiltration was further validated by immunohistochemistry for this exemplary case, with the tumor tissue displaying consistently greater CD4 and CD16 signals compared to normal tissue (Supplementary Figure S24).

In summary, we were able to profile each tumor individually on the protein level based on potential treatment-relevant aberrations in pathway activity, detect divergent expression patterns in special cases and functionally group tumors, e.g. based on immune cell infiltration.

### Personalized expression signatures and treatment recommendations for a prospective MTB case series

**Fig. 5:**
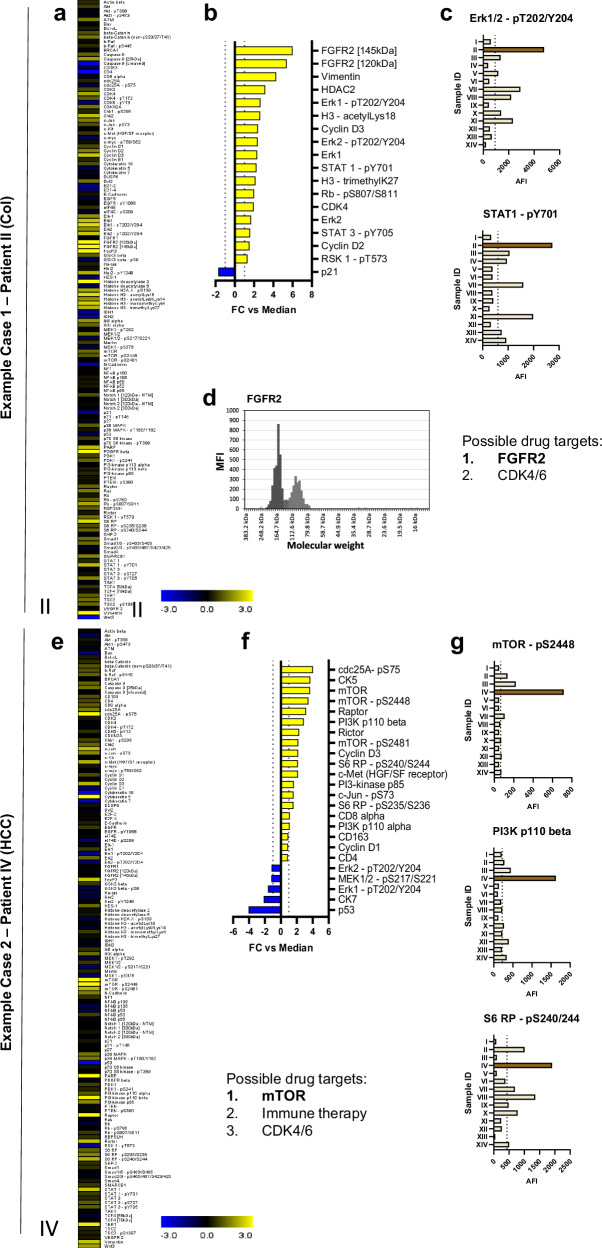
Personalized tumor profiles for select prospective MTB cases. **a**–**d** Case/profile 1 (patient II) – colon carcinoma. **a** Heatmap of DigiWest expression data (normalized AFI) as Log2 FC in relation to median signal (baseline) across all tumors (I-XIV). **b** Selection of key analytes shown relative to baseline signal. **c:** Expression data (normalized AFI) for Erk1/2 – pT202/204 and STAT1 – pY701 in all tumors (I-XIV). Sample-specific signal is shown in brown and dashed line indicates median signal across all samples. **d** DigiWest peak profile of FGFR2. Black peak = 145 kDa, grey peak = 120 kDa; MFI = median fluorescent intensity. **e**–**g** Case/profile 2 (patient IV) – hepatocellular carcinoma. **e** Heatmap of DigiWest expression data (normalized AFI) as Log2 FC in relation to median signal (baseline) across all tumors (I-XIV). **f** Selection of key analytes shown relative to baseline signal. **g** Expression data (normalized AFI) for mTOR – pS2448, PI3K p110 beta and S6 RP – pS240/244 in all tumors (I-XIV). Sample-specific signal is shown in brown and dashed line indicates median signal across all samples. A ranking of potential treatment recommendations based on DigiWest data is given. Most promising drug target printed in bold. Analogue profiles of all other 12 patients are shown in the Supplementary Figs. S26–S37.

In contrast to the analyses discussed above, where archived biobank samples were retrospectively selected, we next aimed at a potential integration of DigiWest into clinical algorithms by investigating tissues gained by a core needle biopsy of tumor tissue. As a proof-of-principle, we included 14 patients who got needle biopsies of their tumors taken as a diagnostic step for the Molecular Tumor Board (MTB) at Tuebingen University. This approach usually does not provide sufficient non-malignant tissue, which was so far used in our investigations to identify tumor-specific up or downregulations. Thus, expression levels from a specific tumor were compared to the “baseline” (median) expression value of a given analyte across all other tumors. The patient cohort of the MTB displayed substantial heterogeneity; it encompassed a variety of gastrointestinal tumor entities, including cholangio-, colon, gallbladder, hepatocellular, pancreatic, gastric, rectal and esophageal carcinomas (Table 2). Furthermore, several patients were heavily pre-treated, in some cases also with targeted therapies. We performed DigiWest analysis using a slightly modified antibody panel of 135 analytes on this small cohort (Supplementary Figure S25). For 12 of the 14 patients, we were able to identify coherent abnormalities regarding the activation of key tumor-related signaling pathways (Table 2), even without normal tissue being available as a reference. Based on our proteomic data, we also identified potential drug targets for each patient (Table 2). Since all patients were included into the MTB, genetic (sequencing) data and respective MTB interpretation was available for each case. Thus, we scrutinized the additional information to this data that could be gained by DigiWest. Upon comparison of proteomic data and MTB treatment recommendation, we observed confirmative pathway alterations to key tumor-driving mutations in 8/12 applicable cases. Two cases are shown in greater detail in Fig. 5. Individual profiles of all other patients are shown in Supplementary Figure S26–37. In the first case of a colon tumor, DigiWest analysis showed elevated expression levels ( > 2-fold) for a multitude of analytes, most notably FGFR2, the phosphorylated variants of STAT1, STAT3, Erk1, and Erk2 along with several cell cycle proteins (Fig. 5a, b). Absolute levels of phospho-Erk and phospho-STAT were the highest among the entire cohort (Fig. 5c) and FGFR2 showed an exceptional DigiWest peak profile (Fig. 5d). Accordingly, the genetic data had identified an amplification of the FGFR2 gene (Table 2). Thus, we were able to prove this observation on the proteomic level, given the notable FGFR2 expression signal well as the activation of downstream pathways MAPK/Erk and Jak/STAT. In the second case, a hepatocellular carcinoma with a peculiar expression signature (Fig. 5e) displayed strongly elevated signals for mTOR-related protein expression and phosphorylation (mTOR, PI3KA, PI3KB, S6 RP, Fig. 5f). Again, signals were substantially higher than in the rest of the cohort (Fig. 5g). On the genetic side, a deletion of the mTOR-regulating tumor suppressor TSC2 was identified (Table 2) confirming the proteomic observations. Furthermore, DigiWest revealed the presence of tumor infiltrating lymphocytes (“hot” tumor) given strong expression levels of CD8 alpha, CD163 and CD4 (Fig. 5f).

**Table 2:** Summary of DigiWest and genetic/MTB data from MTB patient cohort

#	Tumor	Pre-Therapy	MSI Status	Key Deregulated Pathways (DigiWest)	Pathway Identified?	Potential Drug Targets (DigiWest)	Tumor Content (%) - Genetics	Indication of HRD Deficiency?	Key Mutations (MTB-identified)	MTB Drug Recommendation	DigiWest Supportive to MTB?
I	Cholangiocarcinoma	---	stable	cell cycle Histone methylation (DNA Damage)	Yes	1. CDK4/6 2. IDH1 3. Immune therapy	80	no	IDH1 mut Cyclins amp TP53 mut ATM mut	IDH1-inh. (Ivosidenib) FGFR-inh.	(Yes)
II	Colorectal carcinoma	Xgeva/FOLFIRI/ Avastin, XELOX	stable	MAPK/Erk STAT1/STAT3 FGFR2 (Histone modification)	Yes	1. FGFR 2. CDK4/6	80	yes	FGFR2 amp	FGFR-inh. + PD-1/PD-L1	Yes
III	Gallbladder carcinoma	FOLFOX, Gemcitabine/ Cisplatin	stable	PI3K/mTOR Her2/VEGFR2/c-Met (MAPK/Erk)	Yes	1. mTOR 2. Her2 3. TK (Her2/VEGFR/MET) 4. MEK	60	no	BRAF mut CDKN1B/2 A del CDK4 amp	MEK + BRAF-inh. PD1/PD-L1	(Yes)
IV	Hepatocellular carcinoma	Ramucirumab, Cabozantinib, Pembrolizumab	stable	PI3K/mTOR Immune cells cell cycle	Yes	1. mTOR 2. Immune therapy 3. CDK4/6	80	no	TSC2 del	mTOR-inh. PD1/PD-L1 + TKI PD1/PD-L1 + Bevacicumab PD1/PD-L1 + anti-CTLA4	Yes
V	Pancreatic carcinoma	FOLFOX, 5-FU/Onyvide, Gemcitabine/Paclitaxel, FOLFIRINOX	stable	MAPK/Erk PDGFR beta/Her2 (cell cycle)	Yes	1. KRAS 2. PDGFR 3. Her2 4. CDK4/6	80	yes	CDKN1A del KRAS mut	MEK-inh. + CDK4/6-inh. (PARP-inh.)	Yes
VI	Gastric carcinoma (CUP)	Ramucirumab/ Paclitaxel, 5-FU, FLOT, FOLFOX	stable	(Histone acetylation, HDAC6)	No	none	60	no	FAT1 mut NF1 del BRAF mut	TK-inh. (Lenvatinib) + PD1/PD-L1	No
VII	Esophageal carcinoma	FLOT, FOLFIRI	stable	MAPK/Erk Akt/PI3K/mTOR Wnt/beta-Catenin STAT1/STAT3	Yes	1. FGFR 2. TK (PDGFR/VEGFR)	60	yes	FGFR2-PAPSS1 fus BRCA2 mut	FGFR-inh. PARP-inh.	Yes
VIII	Gallbladder carcinoma	---	stable	MAPK/Erk NF-kB Histone acetylation, HDAC	Yes	1. MEK 2. HDAC	80	no	IDH1 mut ARID1A del/mut ATM del/mut BAP1 del/mut	IDH1-inh. PD1/PD-L1	No
IX	Cholangiocarcinoma (metastasis)	---	stable	(IDH1/2, Histone modification)	No	none	50	no	FGFR2-SHTN1 fus BAP1 del/mut	FGFR-inh.	No
X	Pancreatic carcinoma	FOLFOX	stable	MAPK/Erk Wnt/beta-Catenin NF-kB VEGFR2	Yes	1. MEK 2. KRAS 3. VEGFR	n/a	n/a	KRAS mut	MEK-inh. Autophagy-inh.	Yes
XI	Cholangiocarcinoma	Gemcitabine/Cisplatin	stable	p53 loss/cell cycle MAPK/, Akt, STAT Smad signaling PDGFRB, FGFR1	Yes	1. TK (PDGFR/FGFR) 2. CDK4/6 3. TGFBR	25	yes	IDH2 mut SMAD4 mut ARID1A mut (CDKN2A inv)	IDH2-inh.	No
XII	Pancreatic carcinoma	Gemcitabine/ nab-Paclitaxel, mFOLFIRINOX,Gemcitabine/ Erlotinib	stable	cell cycle PI3K/Akt	Yes	1. CDK4/6 2. PI3K 3. mTOR	60	no	TP53 mut KRAS mut	MEK-inh. + CDK4/6-inh.	(Yes)
XIII	Pancreatic carcinoma	mFOLFIRINOXFOLFIRI,Gemcitabine/ nab-Paclitaxel	stable	cell cycle DNA damage Smad signaling	Yes	1. CDK4/6 2. TGFRB	25	no	NBN mut KRAS mut SMAD4 mut	none	n/a
XIV	Rectal carcinoma	FOLFOXIRI, Trifluridin, Tipiracil, Trastuzumab,FOLFIRI/ Panitumumab,Trifluridin/Tipiracil/Bevacizumab	stable	p53 loss/cell cycle PI3K/Akt Her2	Yes	1. CDK4/6 2. Her2 3. PI3K	40	no	TP53 mut ERBB2 amp EGFR amp	none	n/a
					12/14 cases						8 / 12 cases

**Left**: Tumor entity of each included patient (*n* = 14). **Middle**: Key pathways identified by DigiWest are shown. Yes = coherent pathway activity, No = no clear indication of pathway activity. In each case, list of potential drug targets is based solely on DigiWest data. Note: General term “Immune therapy” is used for tumors with notable expression of TIL markers (“hot”); here, any form of immune-related therapy could be considered (e.g. PD1/PD-L1). **Right**: Key mutations as identified by MTB sequencing analysis and MTB treatment recommendation. (mut = mutation, del = deletion, amp = amplification, fus = fusion). Rightmost column: Is DigiWest data confirming potential underlying MTB-identified driver mutations and subsequent drug recommendations? Yes = supportive, (Yes) = partially supportive, No = not supportive. Note: For two cases (XIII and XIV), no MTB drug recommendation was made (n/a).

Thereby, we were able to detect personalized protein signatures in a direct clinical application of DigiWest. For 12/14 cases we identified coherent and treatment-relevant patterns of pathway activation and were able to link protein expression data with genetic mutation analysis and MTB drug recommendations in 8/12 applicable cases, for which complete MTB data was available.

## Discussion

Using ultrasound-guided tissue core biopsies of gastrointestinal tumors, we successfully generated personalized profiles of signal transduction pathway signatures for direct clinical evaluation through high-throughput Western Blotting (DigiWest). Retrospective analysis of primary pancreatic and colorectal tumor tissues relative to normal tissues demonstrated the capabilities of DigiWest to identify treatment-relevant expression signatures for tumor stratification and patient-specific profiling. We then applied the DigiWest system to characterize biopsy-derived tumor samples from current Molecular Tumor Board (MTB) patients in a proof-of-principle approach, testing whether the integration of proteomic data enhances the interpretation of genetic and clinical MTB data to provide a more comprehensive molecular understanding of each tumor. Earlier retrospective analyses of primary tumor tissues of various entities^32,37–39^ have classified and stratified sample groups according to protein expression signatures. In our retrospective analysis, a direct comparison analogue to these previous studies using tumor tissue only revealed tissue-specific markers and entity-relevant pathway activity (e.g. Wnt signaling in colorectal tissues) as described in the literature^40–42^. We expand on this by using patient-matched normal tissues as reference and with this approach our data distinguished colon and pancreatic tumors largely based on the expression of tumor suppressors and oncogenes (p53, Ras, PTEN, p27, Fig. 1c, d) which have commonly described as a frequent mutations in genetic analyses of pancreatic^43,44^ and colorectal carcinomas^45–48^. Thus, our proteomic data comparison mirrors genetic mutations that are different between these two tumor entities. Closer evaluation of the pancreas cohort revealed a division into two subgroups (Fig. 2), There has been extensive evidence of a sub-division of pancreatic carcinoma regarding molecular and clinical phenotypes^49–53^. One particular classification into subtypes^49^ identifies a squamous and immunogenic type, among others. The squamous subtype is for instance characterized by mutations and alterations in TP53 as well as in genes regulating metabolism and autophagy, which matches expression signatures of our Group 1 subtype (p53 reduction, mTOR-related signaling). Group 2 clearly showed upregulation of immune cell markers and Smad signaling, which has been associated with immune responses^54,55^ matching characteristics of immunogenic pancreas carcinomas, which are often regarded as immune-infiltrated. The greater clinical heterogeneity of the colorectal cohort was reflected in our proteomic data. In clinical practice, right- and left-sided colon carcinomas are differentiated^56,57^ as subgroups with strongly different molecular characteristics and treatment responses. For instance, EGFR-directed therapy (EGFR-Ab) is not recommended as a first-line treatment for right-sided colon carcinomas^58^. In line with this, we observed an EGFR downregulation in right-sided carcinomas and an upregulation in left-sided carcinomas. Moreover, there is a general concern among clinical oncologists, that the age of onset for colon carcinomas is decreasing and that early- and late onset tumors greatly differ regarding aggressiveness, treatment options and therapy response^59–61^. Although studies have previously characterized the genetic mutation signatures of early onset colon tumors^62,63^, detailed transcriptional let alone proteomic features have thus far remained elusive^64^.Our data provides potential signaling indicators for patient age and/or tumor localization which might reflect the different clinical behavior of these subgroups and could add important details to improve treatment recommendations, if these observations could be confirmed in larger cohorts. Using DigiWest we were also able to create personalized protein profiles for each individual tumor and identify drug-targetable activity changes in pathway regulation. These personalized characterizations were achieved via the DigiWest approach using only 15 µg of protein, roughly corresponding to a single tissue section. In this specific retrospective study, having matched non-tumorous control tissue was advantageous. However, it is worth noting that differences in tumor/stroma cell content between samples can affect interpretation, as a higher stromal content can partially mask the manifestation of cancer-induced changes in signaling. In line with this, a subsequently performed histopathological evaluation on adjacent tissue sections showed variability in tumor content and tissue section quality (see Supplementary Figure 38). Despite the protein-expression based groupings that were observed across samples (see above), the diversity among samples on an individual level was still evident; we were able accurately depict this thus suggesting a suitability of DigiWest for the analysis of tumors with high clinical and molecular heterogeneity (e.g. #16, #18). Overall, our retrospective analysis also points out a use of DigiWest for direct clinical application, as protein expression and activation on directly druggable targets is achievable on a personalized level with equal sensitivity and high throughput compared to other proteomic methods.

Currently, personalized medicine approaches in clinical oncology still primarily rely on genetic profiling and histological staining, with limited use of high-throughput protein analytics. As a proof-of concept, we analyzed single core needle biopsies from 14 patients with various GI tumors and assessed the protein data complementary to MTB next generation-sequencing (NGS) data. DigiWest profiling data generation proved feasible in this setting, thus adding value to the clinical evaluation through protein analytics. We observed substantial overlap between pathway de-regulations identified by DigiWest and corresponding genetic mutations (e.g. cell cycle activity and *TP53* mutations). Even if there was no direct confirmation of the proteomic data through NGS, as in cases with (partial) concordance between the datasets (such as I, III, IV, V or XII), the observed pathway activation patterns (see Table 2) could still provide additional therapeutic targets. This underscores the importance of accurately detecting treatment-relevant expression signatures and phosphorylation states of druggable signaling proteins and receptors for accurate information transfer on the manifestation of tumor-driving mutations from the genome to the proteome. Furthermore, DigiWest data could also help to identify potential resistance mechanisms since all included MTB patients had experienced relapse. Especially in cases where targeted therapy was applied, the observed changes in expression patterns could (partially) be attributed to resistance development. For example, patient IV (hepatocellular carcinoma) had received treatment with TK-directed inhibitors which could explain the low signal intensities observed for MAPK proteins (see Fig. 5f); thus, elevated mTOR signaling could present a resistance mediator. In a similar approach using RPPA technology, Wahjudi et al. analyzed 27 proteins to retrospectively recommended treatment options for tumor board patients and compared these to genetic information^65^. They noted an inconsistent partial overlap (10-57%) between genetic and proteomic-based recommendations yet emphasized the prognostic value of proteomic data as a readout for tumor physiology and its suitability for integration into precision oncology programs. By the same token, DigiWest is a powerful potential tool to be used complementary and confirmatory to standard sequencing analyses in a clinical setting, with even greater throughput and substantially higher coverage of relevant signaling pathways. This equally holds true for the comparison of DigiWest to multiplexed immunohistochemistry, while also having the advantage of generating semi quantitative data. Furthermore, in a recent publication^66^ we demonstrated good comparability of DigiWest and Mass Spectrometry, however emphasizing a superiority of DigiWest in detecting phosphorylated variants, which is especially important for the question at hand. It can meet the demands of daily clinical routine as it only requires a minimal amount of material (one core needle biopsy obtained during a standard diagnostic procedure) and DigiWest analysis (including data evaluation) can be completed within five days. Moreover, the ability to customize the antibody panel based on specific tumor types or to align with particular drug targets is highly advantageous. DigiWest can provide additional insights into treatment options and facilitate appropriate drug selection on a personalized level, thereby expanding the basis of information available to the oncologist. Methodologically, it aids in distinguishing relevant tumor drivers and selecting the most suitable treatment for individual patients, enhancing the success rate of targeted therapies and ultimately improving treatment outcome. Notably, both our retrospective and prospective datasets demonstrate the potential of the method. Even if no matched reference tissue is available, increasing cohort sizes would stabilize baseline expression values by counteracting variability introduced by tissue heterogeneity and variations in tumor content to ultimately serve as a reliable reference in clinical practice. It is worth noting that the accuracy of DigiWest data is improved even more by including patient-matched normal tissue which could be implemented by sampling an additional biopsy. These findings suggest incorporating DigiWest profiling into future clinical studies to evaluate the potential of this technology to further improve response prediction.

## Methods

### Retrospective patient cohort

A total of 20 tumors (10 pancreatic and 10 colorectal carcinomas each) was utilized and available samples were selected from the tumor bank of the University Hospital Tübingen. Research involving human research participants, material, or data were performed in accordance with the Declaration of Helsinki. The patients gave written informed consent and the study was reviewed and approved by the local ethics committee at the medical faculty of the University of Tübingen (364/2023BO2). In addition, only tumors for which non-tumorous, normal tissue from the same patient was available, were included. All tumor (*n* = 20) and normal tissues (*n* = 20) were obtained as fresh-frozen samples. At the time of surgery, none of the patients with pancreatic tumors had previously received any systemic anticancer treatment, whereas four of the colon carcinoma patients did. All other clinical information available for these tumors, which also includes MSI status, is shown in Table 1. For the protein analysis, layered cuts of 10 µm each from the tissue blocks were prepared for each sample (pancreas tissues: 20 curls/sample, colon tissues: 15 curls/sample). All tumor and normal tissues were subjected to a subsequent, retrospective histological analysis. Representative H&E tissue sections for all samples are shown in Supplementary Figure S38). The pathologist´s comments on the analyzed sections regarding tumor content, tissue quality and heterogeneity are included in the figure legend. Genetic profiling was not performed on this cohort.

### Prospective analysis of biopsy samples (MTB patient cohort)

Tumor samples for DigiWest analysis were obtained from patients that received a core needle biopsy to perform NGS analysis for the MTB (*n* = 14). Research involving human research participants, material, or data were performed in accordance with the Declaration of Helsinki. The patients gave written informed consent, and the study was reviewed and approved by the local ethics committee at the medical faculty of the University of Tübingen (341/2021BO2). Details on tumor tissue origin, patient age and previous therapy are shown in Table 2. Samples were stored at −80 °C until lysis.

### Sample preparation for DigiWest

Before protein profiling analysis, tissue sections (curls) were lysed using 50 µl of lysis buffer (LDS Lysis Buffer (Life Technologies, Carlsbad, CA, USA), supplemented with 10% reducing agent (Thermo Fisher Scientific), 4% Protease-Inhibitor (Roche, Basel, Switzerland) and 10% Phosphatase-Inhibitor (Roche)). Proteins were denatured by heating to 95 °C for 10 min. Fresh frozen biopsy samples were lysed with 50–100 µl of lysis buffer and homogenized using a pistil during heating.

For all utilized samples, protein quantification was performed using in-gel staining. 1 µl of each original lysate per sample were loaded onto a NuPAGE 4–12% Bis-Tris precast gel (Thermo Fisher Scientific) and run according to the manufacturer´s instructions. The gel was washed with water and proteins were stained with BlueBandit (VWR, Radnor, PA, USA) for 1 h. The gel was de-stained over night with ddH2O before detection on a LI-COR Odyssey instrument. Analysis and protein quantification was performed using ImageStudio and signals were compared to reference samples of known protein amounts.

### DigiWest protein profiling

DigiWest was performed as published^31^ using 15 µg of cellular protein. In brief, the NuPAGE system (Life Technologies) was used for gel electrophoresis and blotting onto PVDF membranes. Proteins were biotinylated on the membrane using NHS-PEG12-Biotin (50 µM) in PBST for 1 h. Sample lanes were cut into 96 strips (0.5 mm each) and placed in one well of a 96-well plate before adding 10 µl elution buffer (8 M urea, 1% Triton-X100 in 100 mM Tris-HCl pH 9.5). Each strip/protein fraction was incubated with 1 distinct Neutravidin-coated MagPlex bead population (Luminex, Austin, TX, USA). Coupling was performed over-night and non-bound binding sites were blocked with 500 µM deactivated NHS-PEG12-Biotin for 1 h. By pooling all 96 protein-loaded bead populations, the original sample lane was reconstituted.

5 µl aliquots of bead mix were added to 96-well plates containing 50 µl assay buffer (Blocking Reagent for ELISA (Roche) supplemented with 0.2% milk powder, 0.05% Tween-20 and 0.02% sodium azide). Upon discarding of the assay buffer, 30 µl of primary antibody (diluted in assay buffer) was added per well. After overnight incubation at 15 °C, the bead-mixes were washed twice with PBST and species-specific PE-labelled (Phycoerythrin) secondary antibodies (Dianova, Hamburg, Germany) were added for 1 h at 23 °C. Beads were washed twice with PBST before readout on a Luminex FlexMAP 3D instrument.

136 (retrospective analysis of pancreas and colon carcinomas) or 132 (non-retrospective analysis of GI tumors) primary antibodies (Supplementary Data 1) were selected from a collection of >1500 available antibodies, all of which are performance-evaluated and routinely used in DigiWest. Pathway allocation of analytes was mapped based on the Kyoto Encyclopedia of Genes and Genomes (KEGG) database^67,68^. Analyte selection for the retrospective analysis was largely based on KEGG entries for pancreatic cancer (hsa05212) and colorectal cancer (hsa05210) and previous experience with the analysis with these tissue/sample types. The panel was slightly altered for the prospective study to also include proteins commonly affected in other GI tumors.

Peak identification and integration were performed using an Excel-based analysis tool. For the retrospective analysis, a total of 150 peaks were identified, with 137 (91.3%) generating reliable and non-weak signals (AFI > 50). For the prospective analysis, good signal was detected for 135 / 142 identified peaks (95.1%). For all samples, signal intensity was normalized to total protein amount loaded onto the beads. The software package MeV 4.9.0 was used for heatmap generation and differential expression analysis^69^. Hierarchical clustering (HCL) was performed using Euclidian Distance and complete linkage. For heatmaps using absolute expression data (Fig. 1**+** Fig. 5), fluorescent signals were median centered across samples for a given analyte and Log2-transformed. In the MTB patient cases, median fluorescent signal was calculated across all 14 samples for each analyte, which was regarded as baseline expression value; tumor-specific signals were subsequently set in relation to baseline (Log2 Fold Change). For all relative data, (Fig. 2**-** Fig. 4) Log2 Fold Changes of expression signals against the respective matched normal tissue were calculated for each analyte and directly used for heatmap generation. The entire DigiWest dataset is shown in Supplementary Figure S1 and all raw and normalized DigiWest data can be found in Supplementary Data 2-3.

### Genetic analysis of prospective MTB patient cohort

All tumors from patients of the prospective MTB-cohort received next generation panel sequencing by CeGaT GmbH, Tübingen or the Institute of Medical Genetics and Applied Genomics, Tübingen as previously described^70^. The identified therapy-relevant alterations and the drug recommendations of the MTB for each patient are shown in Table 2.

### Statistical analysis

Statistical analysis was performed using GraphPad Prism 9 and 10 (Graphpad Software, San Diego, CA, USA). Data was tested for normality using the Shapiro-Wilk test. Only if groups were normally distributed, they were compared via unpaired, two-tailed t-test. If normality was not met, the two-tailed Mann-Whitney-U Test was used. The Wilcoxon Rank-Sum test was used for differential expression analysis (heatmaps). N numbers and further statistical details for each experiment can be found in the respective figure legend. In all cases, a *p* value < 0.05 was considered significant unless stated otherwise and all exact p-values are stated in the respective figures.

## Supplementary information

Supplementary Information

Supplementary Data 1

Supplementary Data 2

Supplementary Data 3

## Data Availability

All DigiWest-related raw and normalized data generated or analyzed during this study are included within the article (see **Supplementary Data 2–3**).Our ethical approval does not allow the complete upload of the results from patient DNA sequencing. All relevant information from the DNA sequencing are included in the manuscript. All other datasets used and/or analyzed during the study are available from the corresponding author on reasonable request.
