# Supplementary material for: Personalized signaling pathway analysis of gastrointestinal tumors for patient stratification and drug target evaluation using clinically derived core biopsies
Source: NPJ Precis Oncol. 2026 Feb 25;10:124. doi: 10.1038/s41698-026-01304-5 (PMC13003121; doi:10.1038/s41698-026-01304-5)
Supplement: Supplementary file 1 — Supplementary Information [file 41698_2026_1304_MOESM1_ESM.pdf]

Personalized signaling pathway analysis of gastrointestinal tumors for patient stratification and drug target evaluation using clinically derived core biopsies

Stahl et al.

Supplementary Information

I. Supplementary Figures

II. Supplementary Tables

I. Supplementary Figures

Suppl. Fig. S1

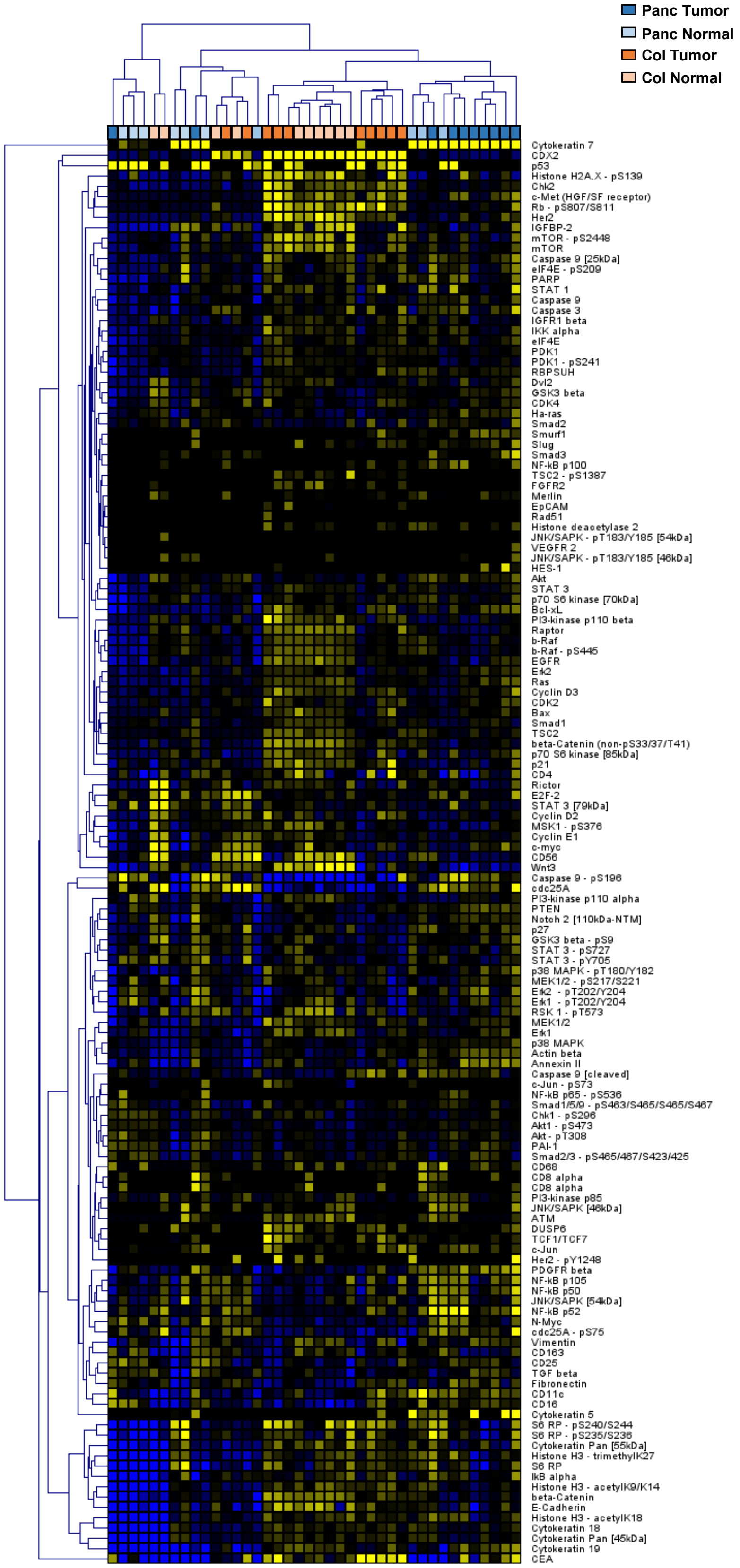

Suppl. Fig. S1: Heatmap and Hierarchical Cluster analysis of DigiWest data set including all retrospectively analyzed tumor and normal (non-tumorous) tissue samples (n = 40). Clustering was performed using Euclidian Distance and complete linkage.

Suppl. Fig. S2

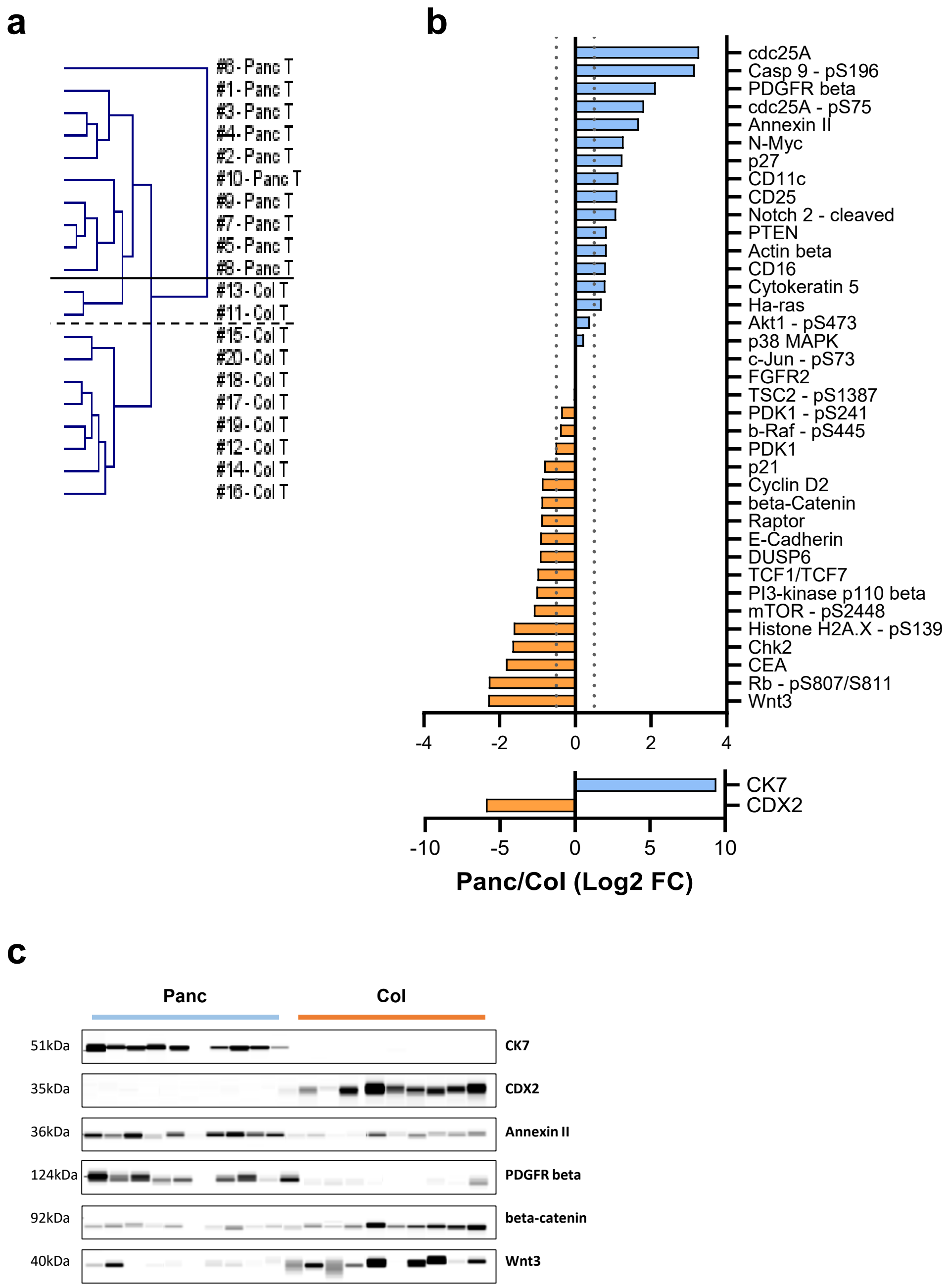

Suppl. Fig. S2: a: Hierarchical Cluster analysis of all pancreas and colon tumor tissues (n = 20). Clustering was performed using Euclidian Distance and complete linkage. Solid line indicates separation based on tissue type; dashed line based on clustering only. **b:** Log2 Fold changes (Median AFI Panc/ Median AFI Col) of all differentially expressed analytes. Analytes with higher expression in pancreas tumors indicated in blue, colon in orange. **c:** Western Blot mimics (grayscale maps generated from DigiWest data) of selected differentially expressed proteins. For graphical representation, background-subtracted raw data from representative pancreas and colon samples were used.

Suppl. Fig. S3

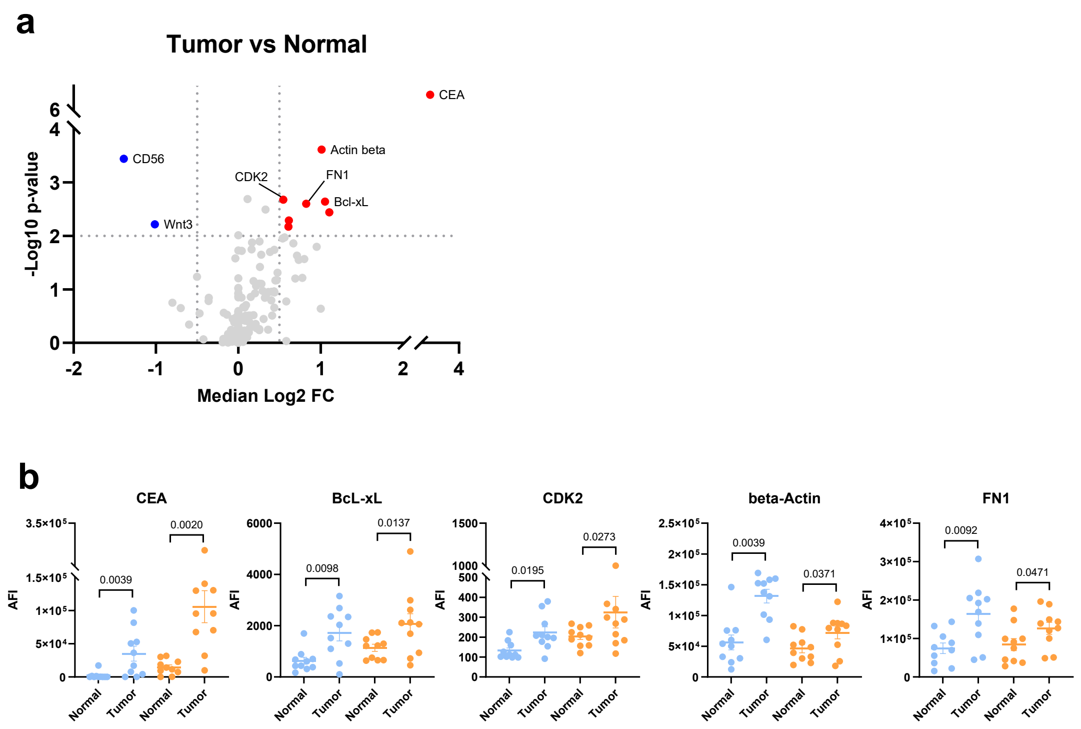

Suppl. Fig. S3: a: Volcano plot of comparison between all tumor (n=20) and their respective normal tissues (n=20); paired t-test, $p < 0.01$. Significantly upregulated proteins are shown in red, downregulated proteins in blue. Analytes with FCs < I0.5I are excluded. **b:** DigiWest data (normalized AFI) for analytes with tumor-associated effects consistent in both tumor types. Separate tumor versus normal comparison for each tumor type (blue = pancreas, orange = colon, tumor - n=10 each, normal – n=10 each); p-value as indicated. Solid line indicates the mean FC value per group. Error bars: S.E.M.

Suppl. Fig. S4

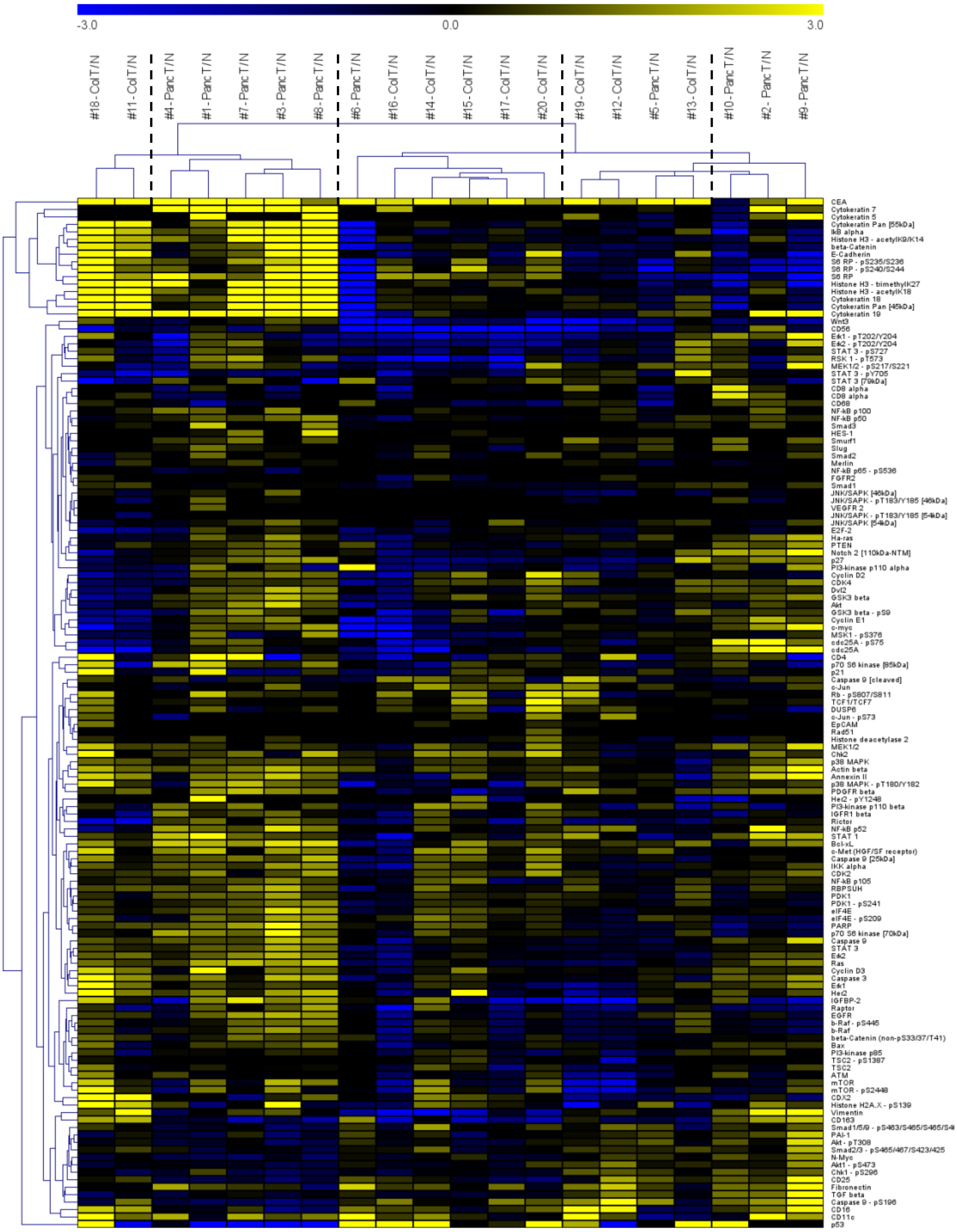

Suppl. Fig. S4: Hierarchical Cluster Analysis of all tumor tissues (n = 20) in relation to their respective, patient-matched normal tissue (T/N as Log2 Fold Changes). Dashed lines separate the observed four sample clusters. Clustering was performed using Euclidian Distance and complete linkage.

Suppl. Fig. S5

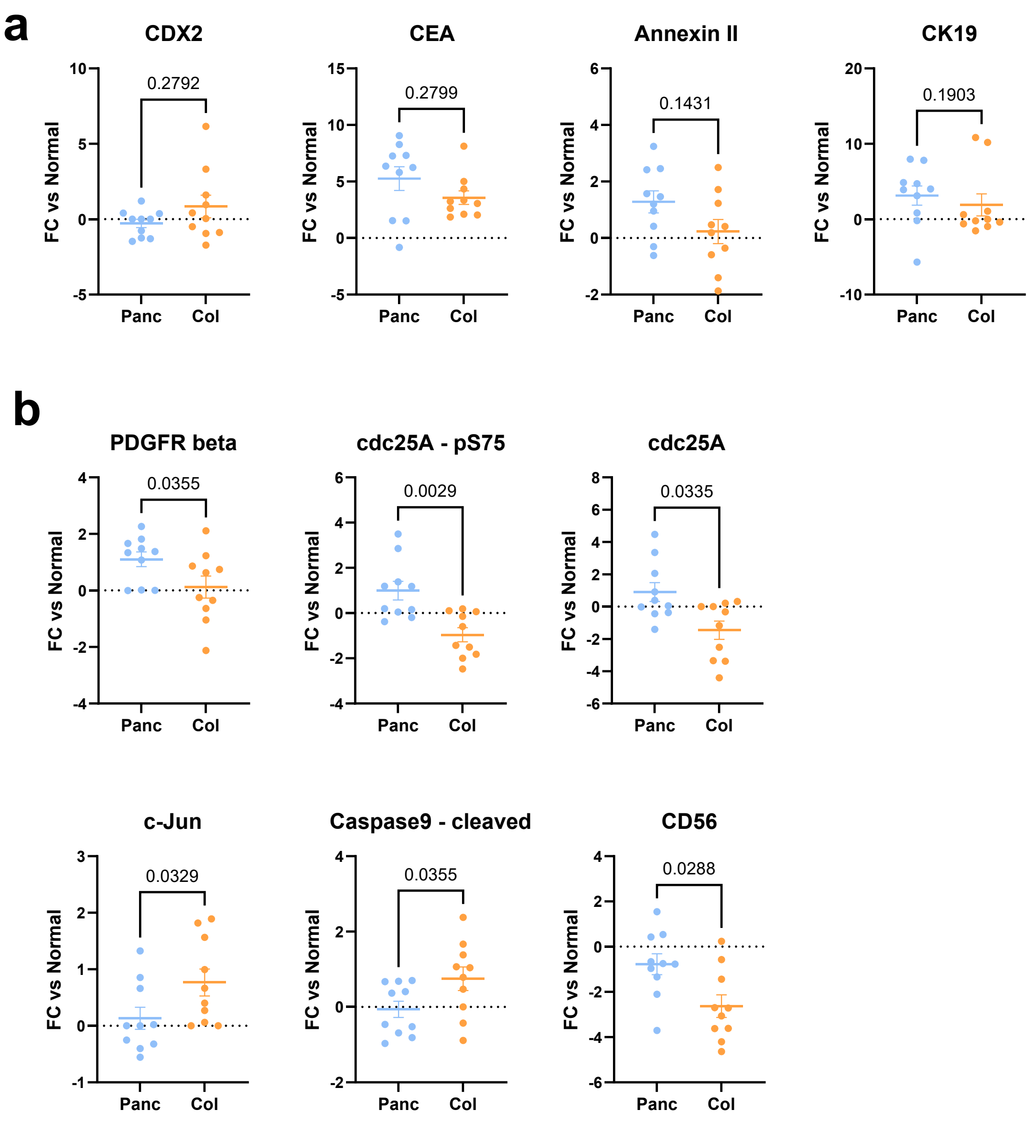

Suppl. Fig. S5: Tumor/normal-matched relative DigiWest data (as Log2 FCs) for additional differentially expressed analytes between tumor types (n = 10 each) based on the comparison in **Fig. 1d**; Mann-Whitney test, $p < 0.05$. **a:** Tissue-specific markers mostly show no differential effects. **b:** Analytes including proteins stemming from tumor suppressor- and oncogenes showing significant differences. Panc = Pancreas (blue), Col = Colon (orange); p-value as indicated. Solid line indicates the mean FC value per group. Error bars: S.E.M.

Suppl. Fig. S6

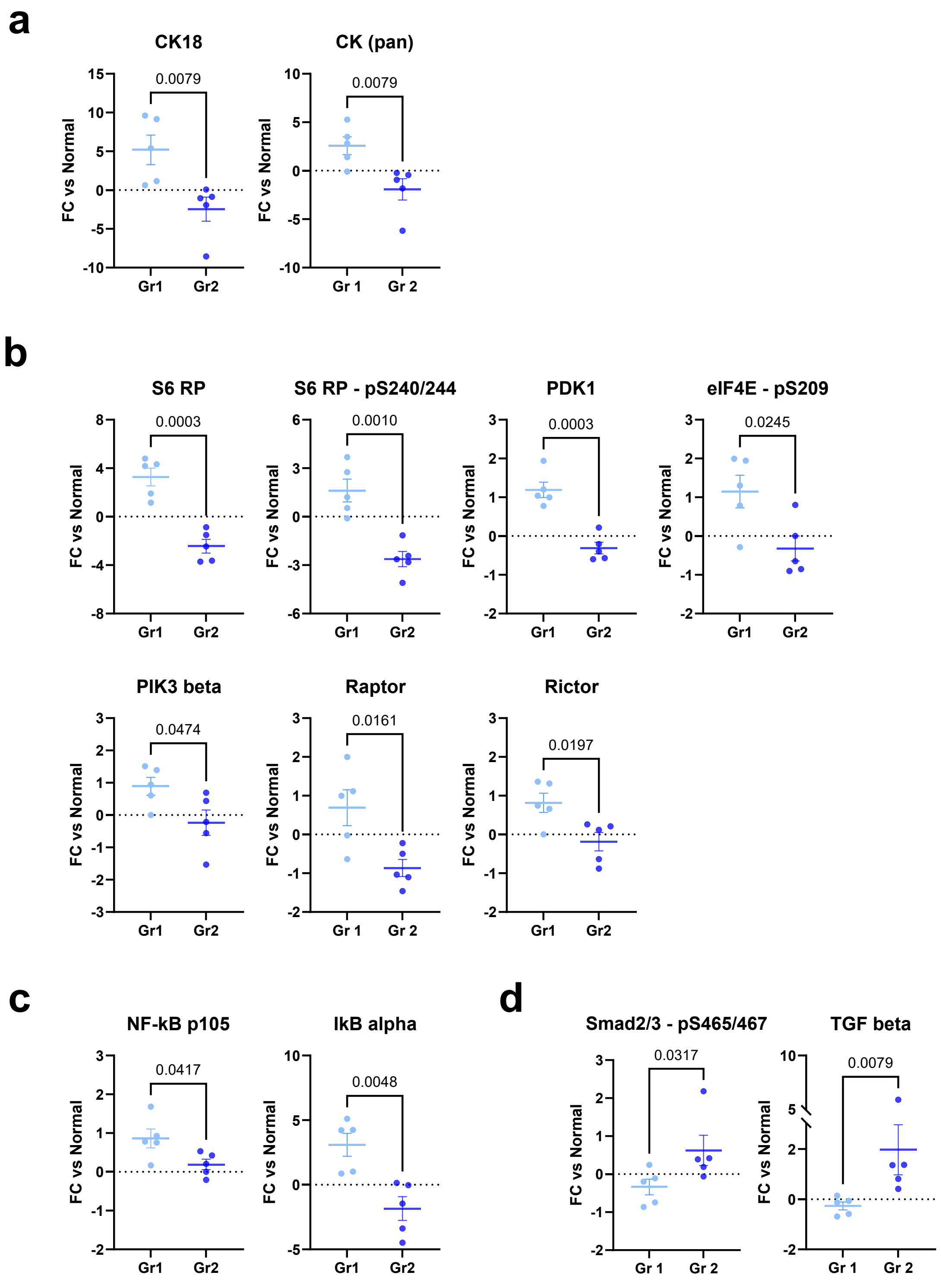

Suppl. Fig. S6: Tumor/normal-matched relative DigiWest data (as Log2 FCs) for additional differentially expressed analytes between Group 1 (light blue, n = 5) and Group 2 (dark blue, n = 5) pancreas tumors based on the comparison from **Fig. 2c-e**; Mann-Whitney test, $p < 0.05$. **a-d:** Analytes of interest sorted according to their pathway/function allocation. **a:** Cytokeratins, **b:** mTOR signaling/protein synthesis, **c:** NF-kappaB signaling, **d:** immune cell markers/Smad signaling. Data shown is a larger collection of relevant analytes from **Fig. 2c-e.** Either the Mann-Whitney test or unpaired t-test was used depending on data distribution; p-value as indicated. Solid line indicates the mean FC value per group. Error bars: S.E.M.

Suppl. Fig. S7

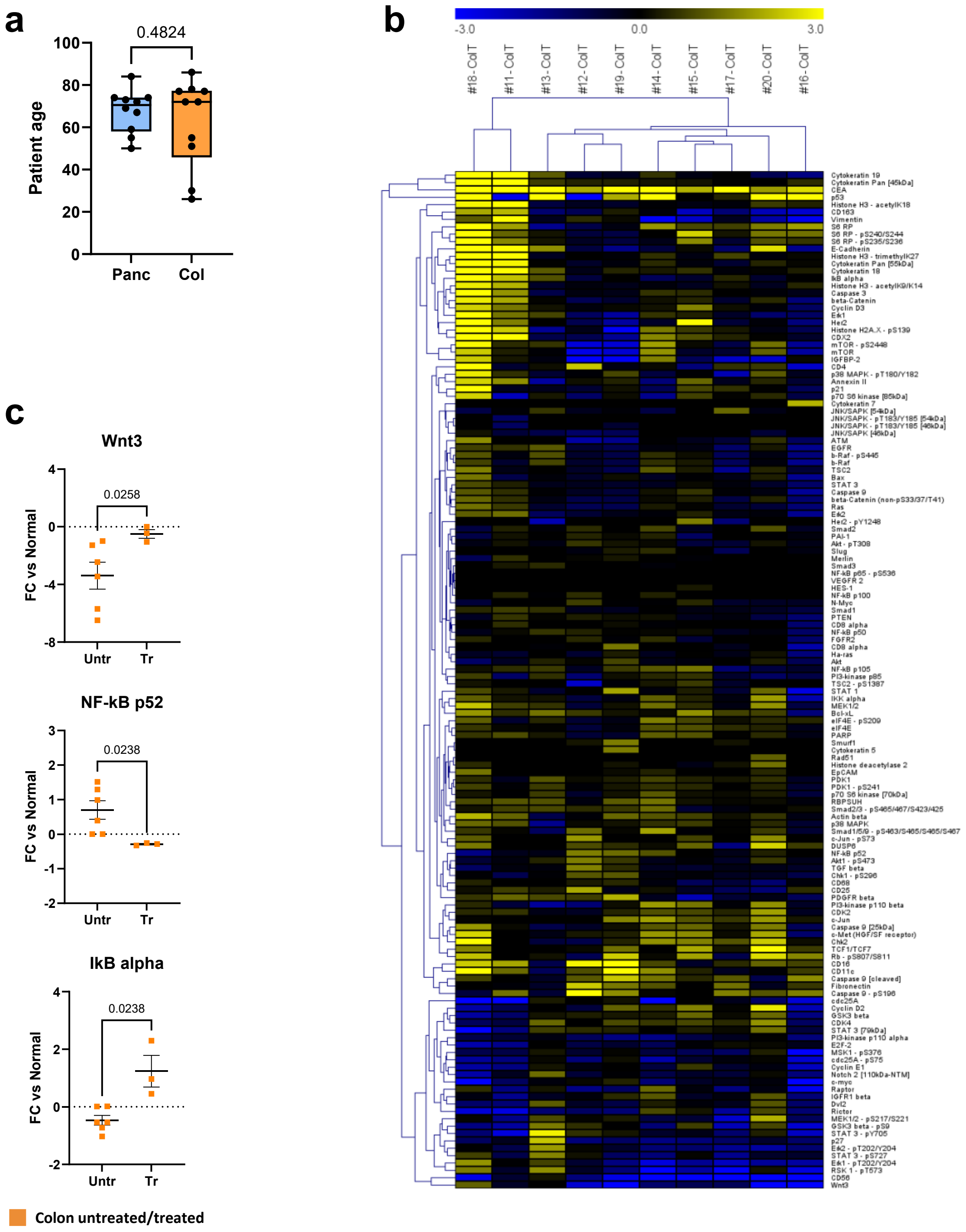

Suppl. Fig. S7: a: Boxplots showing distribution of patient age in pancreas (blue) and colon (orange) CA cohorts (n = 10 each); unpaired t-test. Solid line within the box indicates average patient age/group with whiskers indicating the range (min/max). Individual patient ages are also shown in **Table 1**. **b:** Hierarchical Cluster Analysis of colon tumor tissues (n = 10) in relation to their respective, patient-matched normal tissue (T/N as Log2 Fold Changes). Clustering was performed using Euclidian Distance and complete linkage. Sample tree is also shown in **Fig. 3a**. **c:** Tumor/normal relative DigiWest data (Log2 FCs) for differentially expressed analytes comparing untreated (n = 6) and pre-treated (n = 3) colorectal tumors. Due to differences in group size, Welch´s t-test ($p < 0.05$) was used instead of unpaired t-test. p-value as indicated. Solid line indicates the mean FC value per group. Error bars: S.E.M.

Suppl. Fig. S8

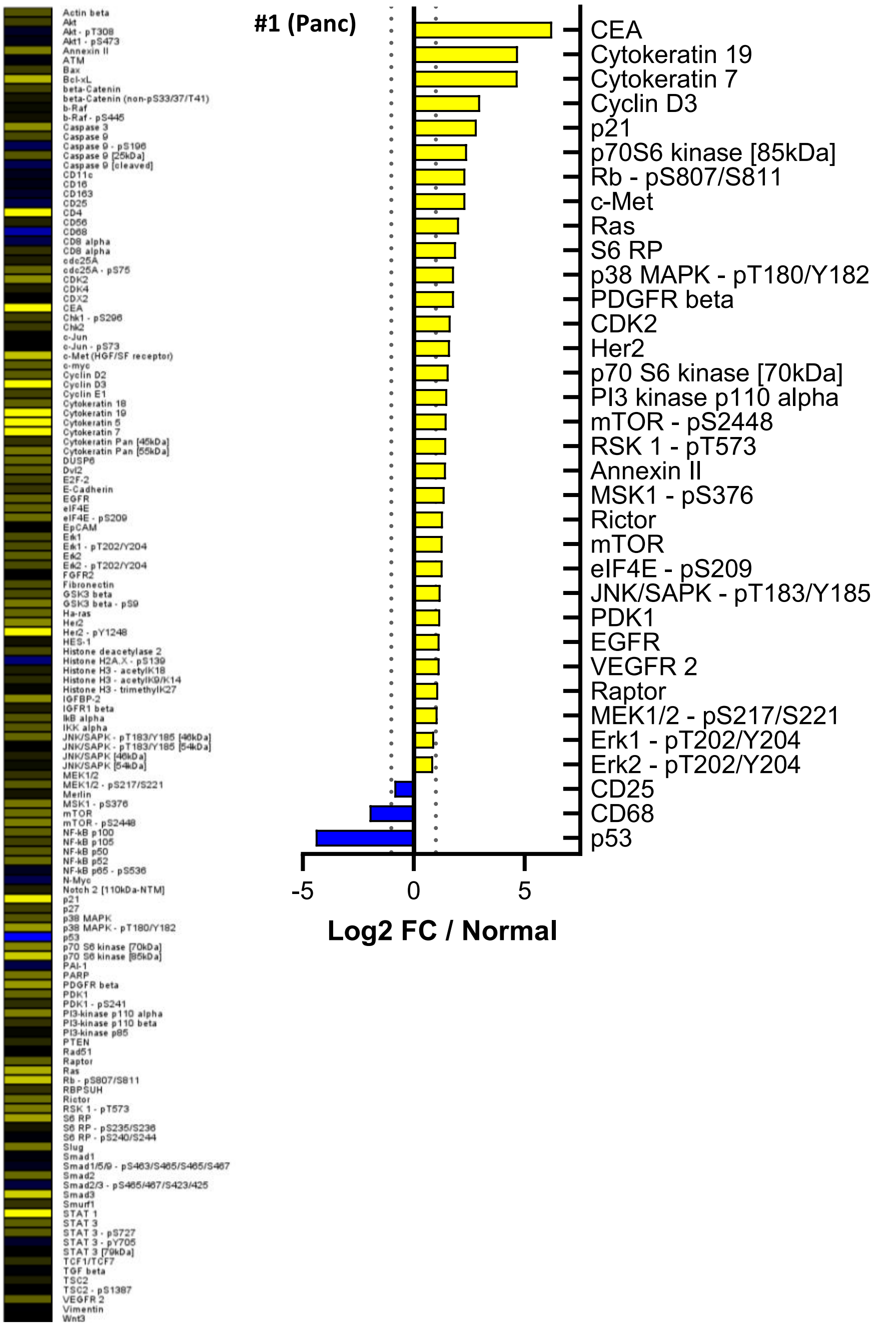

Suppl. Fig. S8: Individual protein profile of pancreas tumor #1. Heatmap showing tumor/normal relative DigiWest data (Log2 FCs) of all analytes (left) and election of key up- or downregulated analytes versus matched normal tissue (right). Selection was based on markers and key regulatory (pathway) proteins.

Suppl. Fig. S9

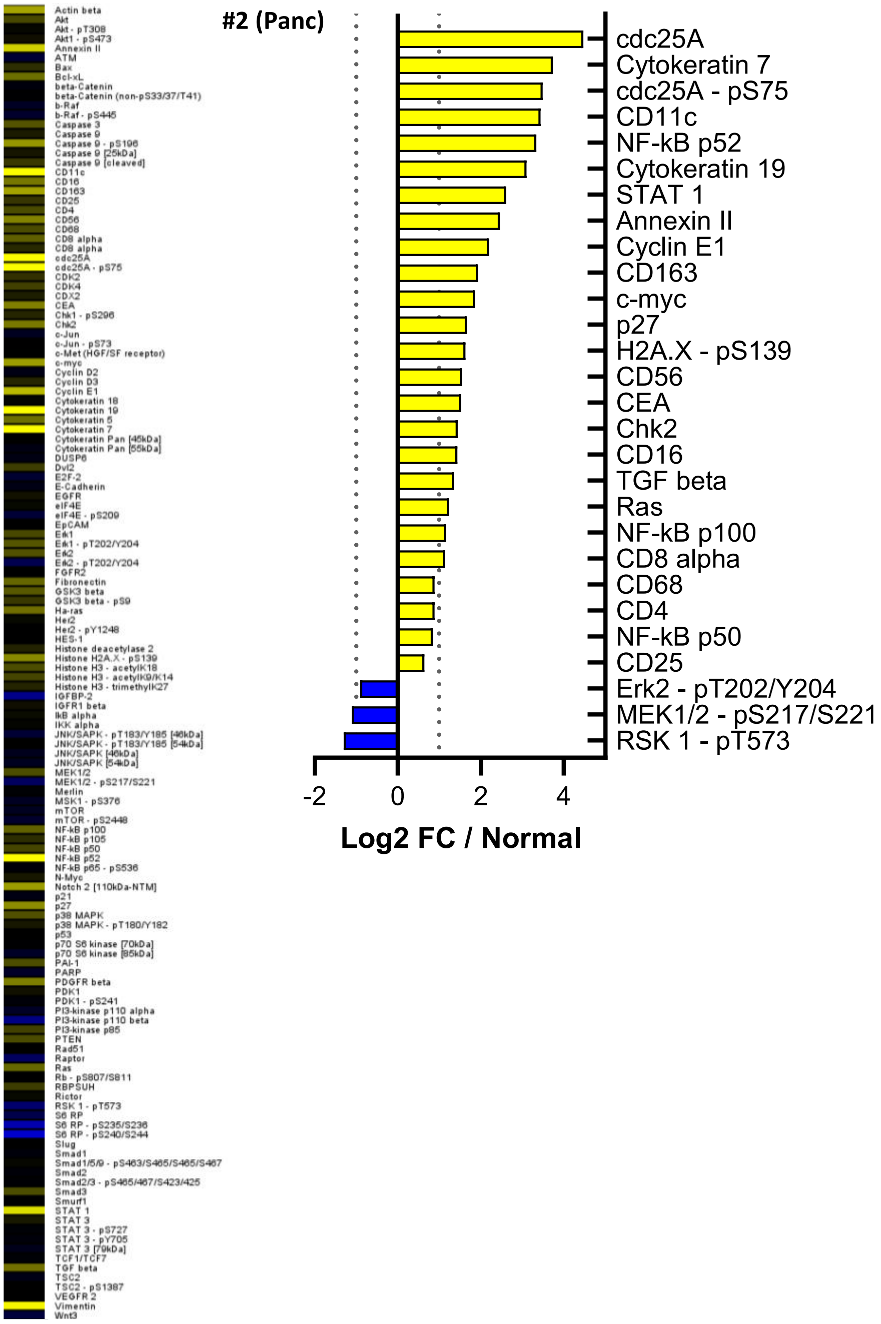

Suppl. Fig. S9: Individual protein profile of pancreas tumor #2. Heatmap showing tumor/normal relative DigiWest data (Log2 FCs) of all analytes (left) and election of key up- or downregulated analytes versus matched normal tissue (right). Selection was based on markers and key regulatory (pathway) proteins.

Suppl. Fig. S10

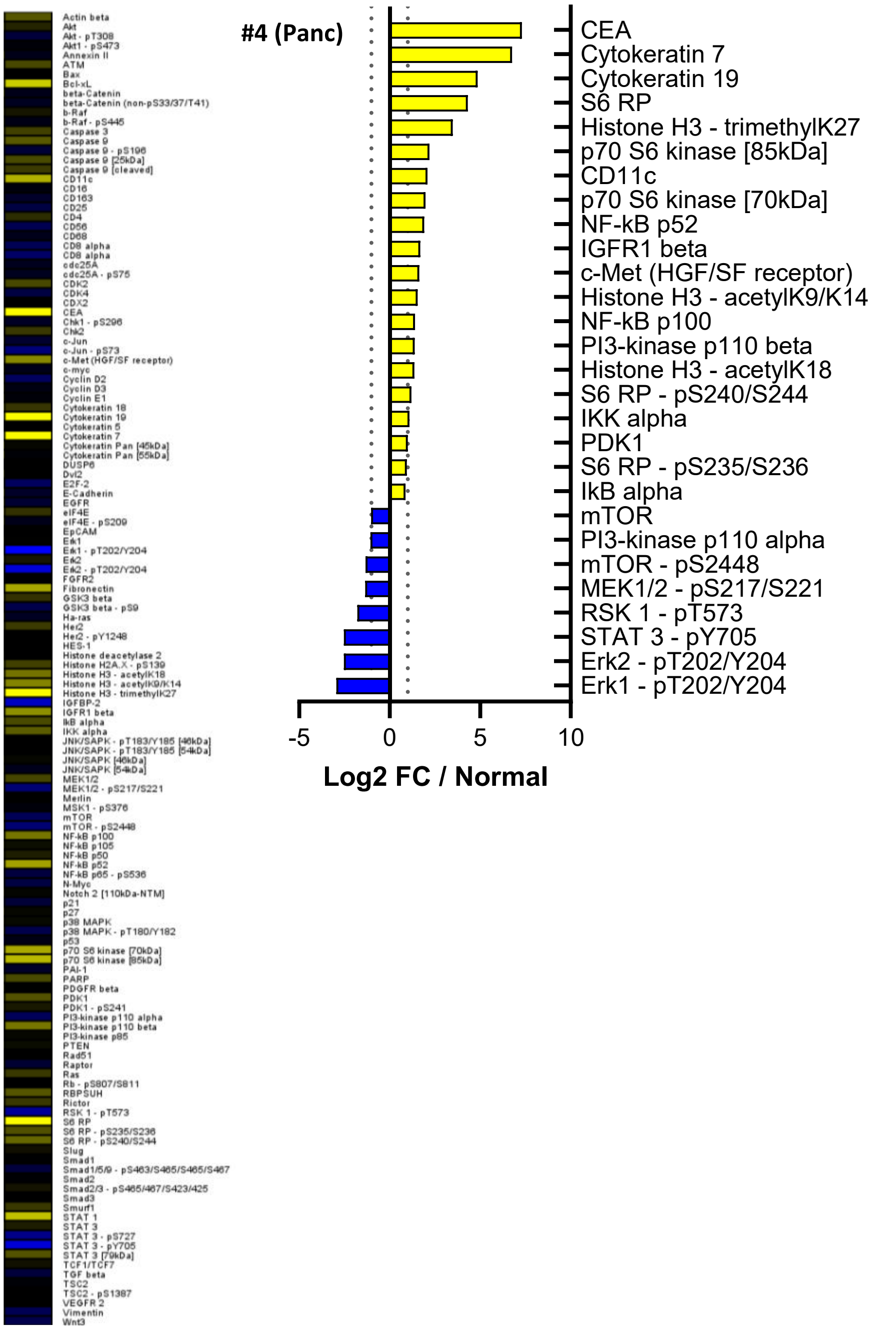

Suppl. Fig. S10: Individual protein profile of pancreas tumor #4. Heatmap showing tumor/normal relative DigiWest data (Log2 FCs) of all analytes (left) and election of key up- or downregulated analytes versus matched normal tissue (right). Selection was based on markers and key regulatory (pathway) proteins.

Suppl. Fig. S11

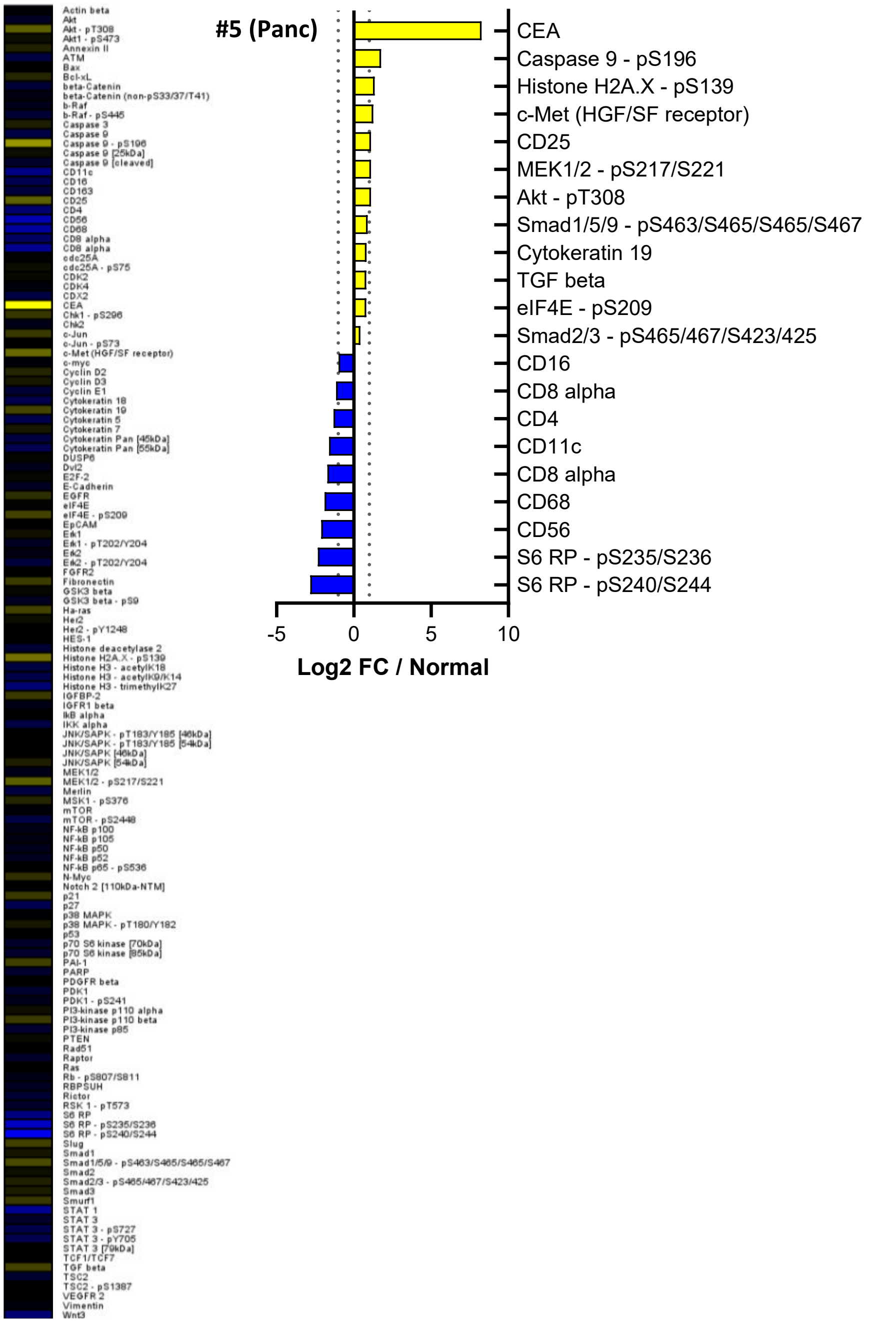

Suppl. Fig. S11: Individual protein profile of pancreas tumor #5. Heatmap showing tumor/normal relative DigiWest data (Log2 FCs) of all analytes (left) and election of key up- or downregulated analytes versus matched normal tissue (right). Selection was based on markers and key regulatory (pathway) proteins.

Suppl. Fig. S12

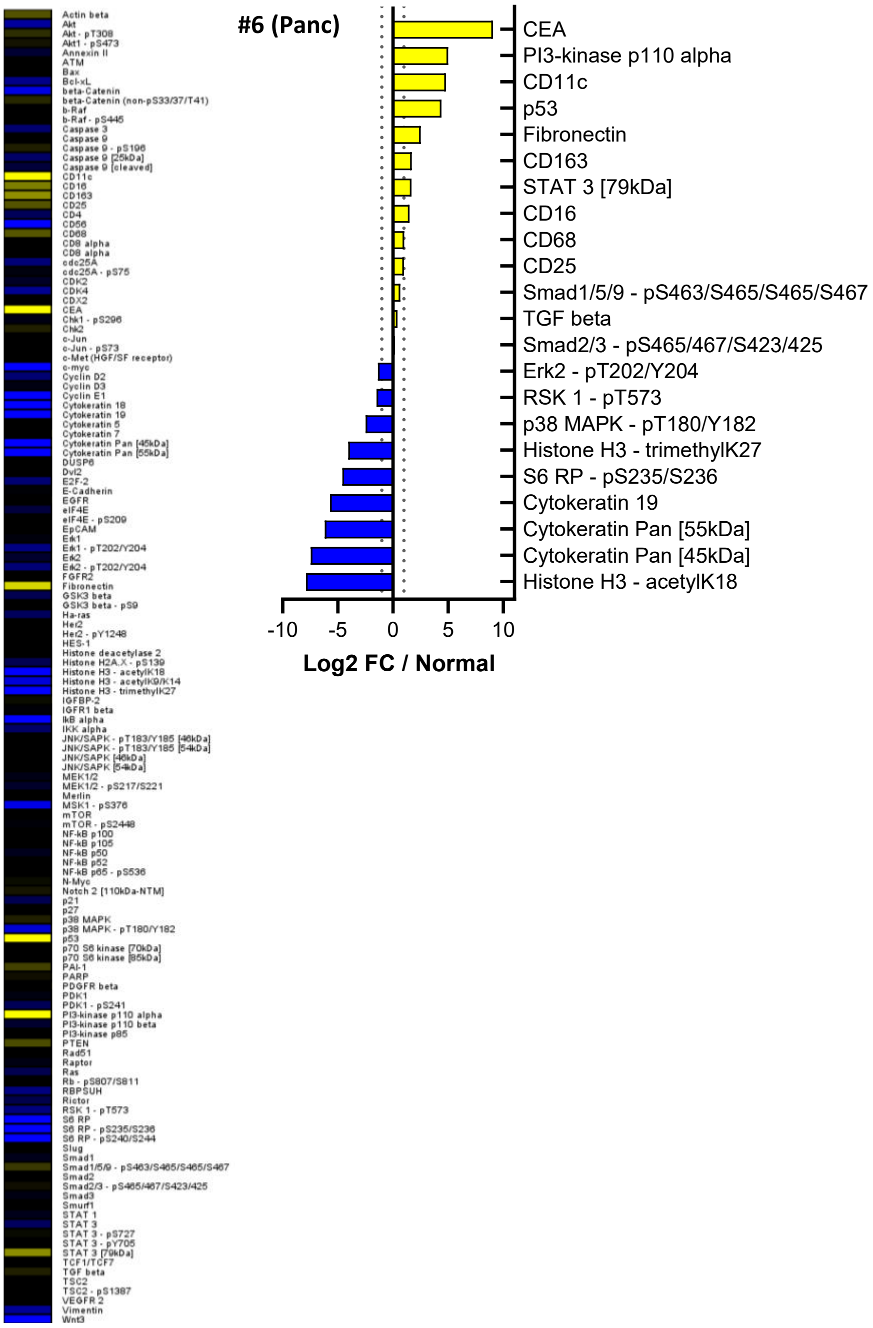

Suppl. Fig. S12: Individual protein profile of pancreas tumor #6. Heatmap showing tumor/normal relative DigiWest data (Log2 FCs) of all analytes (left) and election of key up- or downregulated analytes versus matched normal tissue (right). Selection was based on markers and key regulatory (pathway) proteins.

Suppl. Fig. S13

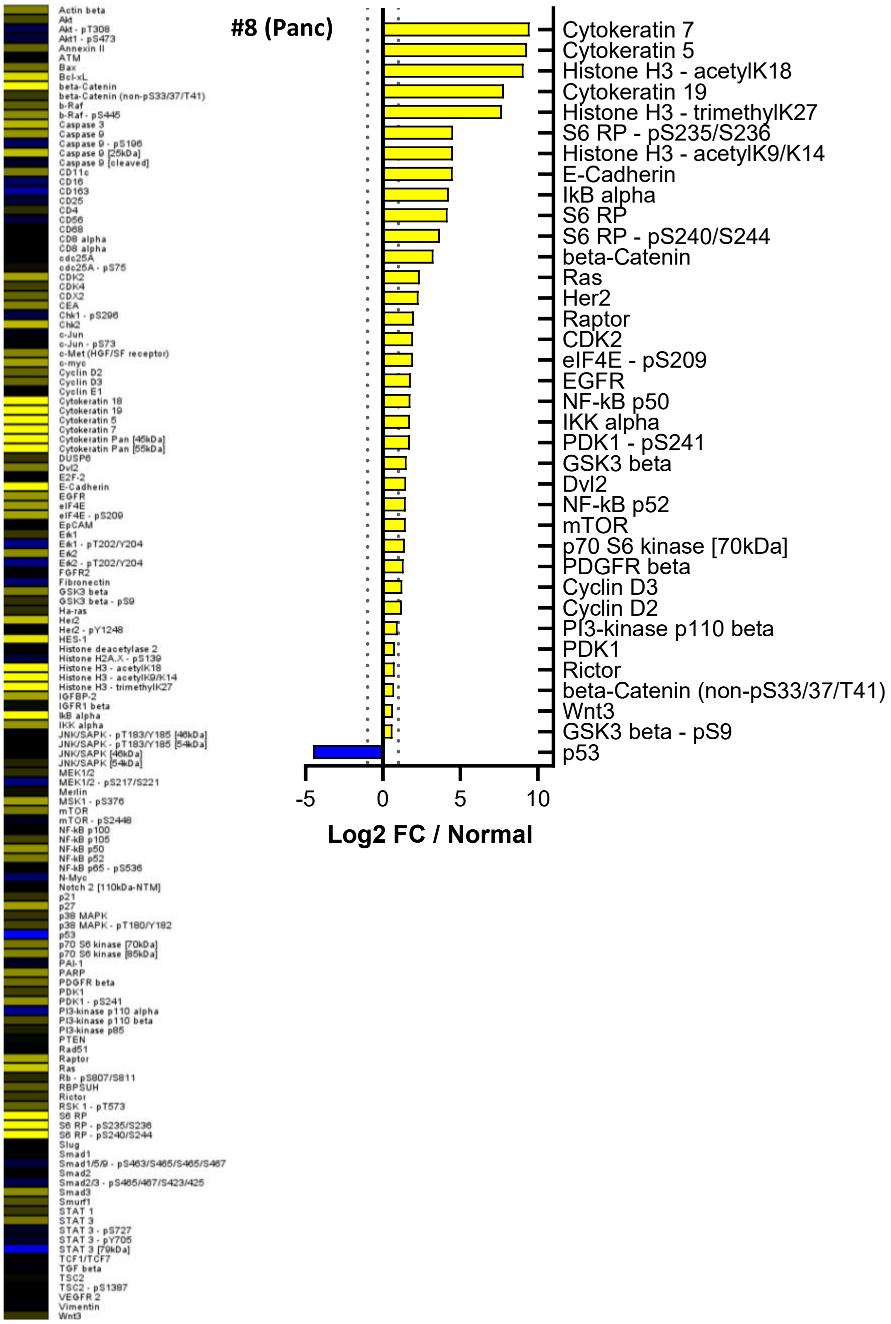

Suppl. Fig. S13: Individual protein profile of pancreas tumor #8. Heatmap showing tumor/normal relative DigiWest data (Log2 FCs) of all analytes (left) and election of key up- or downregulated analytes versus matched normal tissue (right). Selection was based on markers and key regulatory (pathway) proteins.

Suppl. Fig. S14

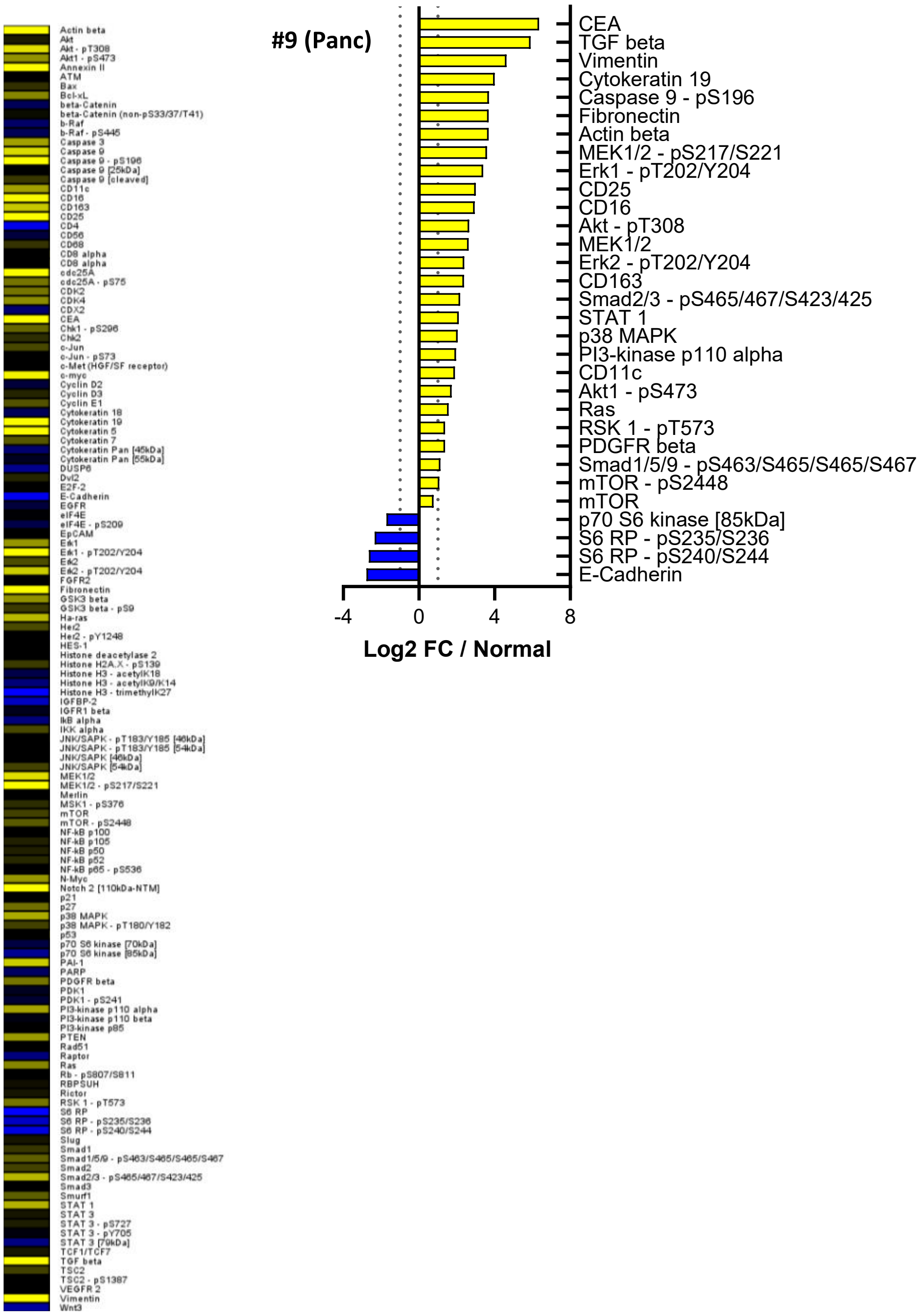

Suppl. Fig. S14: Individual protein profile of pancreas tumor #9. Heatmap showing tumor/normal relative DigiWest data (Log2 FCs) of all analytes (left) and election of key up- or downregulated analytes versus matched normal tissue (right). Selection was based on markers and key regulatory (pathway) proteins.

Suppl. Fig. S15

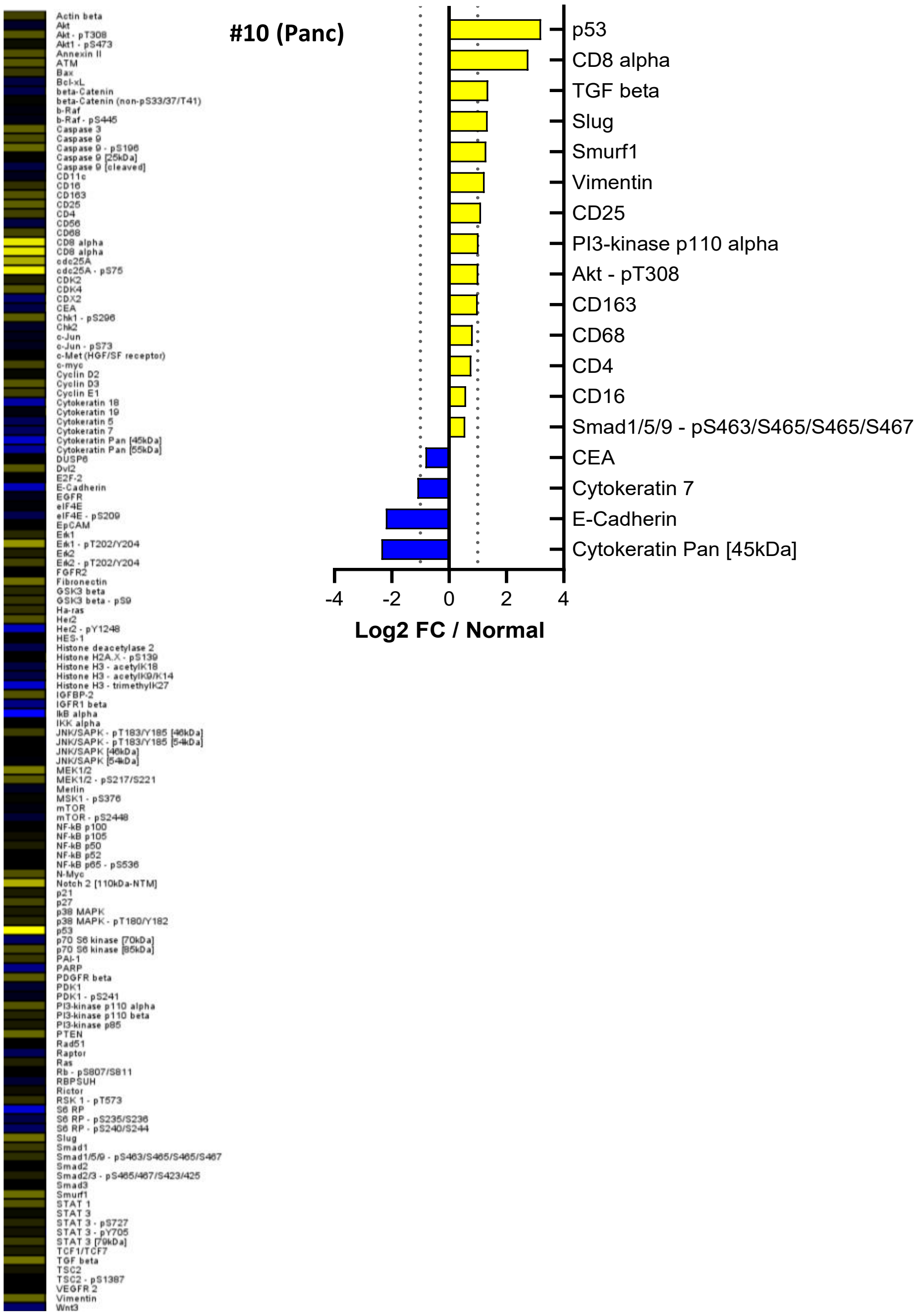

Suppl. Fig. S15: Individual protein profile of pancreas tumor #10. Heatmap showing tumor/normal relative DigiWest data (Log2 FCs) of all analytes (left) and election of key up- or downregulated analytes versus matched normal tissue (right). Selection was based on markers and key regulatory (pathway) proteins.

Suppl. Fig. S16

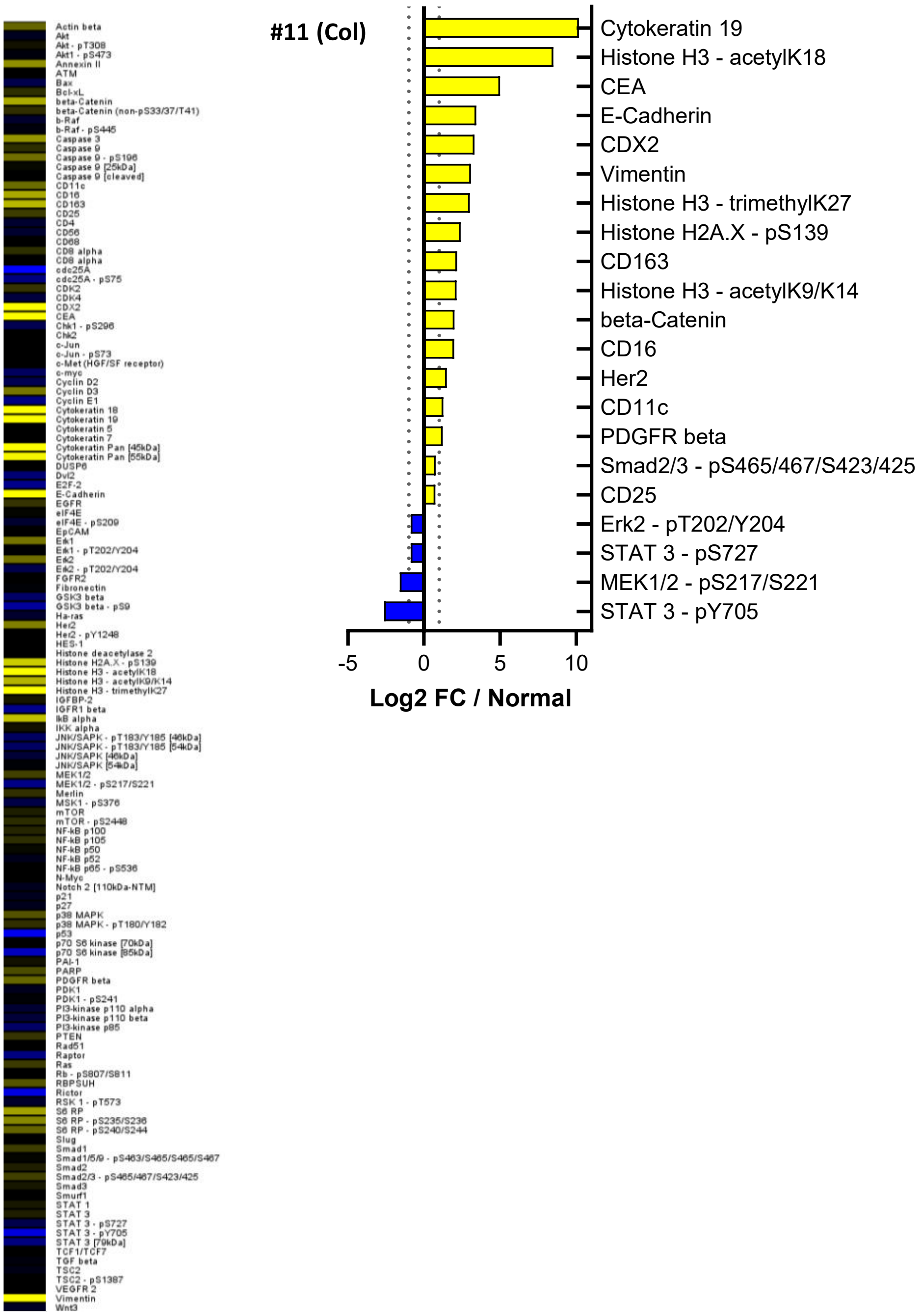

Suppl. Fig. S16: Individual protein profile of colon tumor #11. Heatmap showing tumor/normal relative DigiWest data (Log2 FCs) of all analytes (left) and election of key up- or downregulated analytes versus matched normal tissue (right). Selection was based on markers and key regulatory (pathway) proteins.

Suppl. Fig. S17

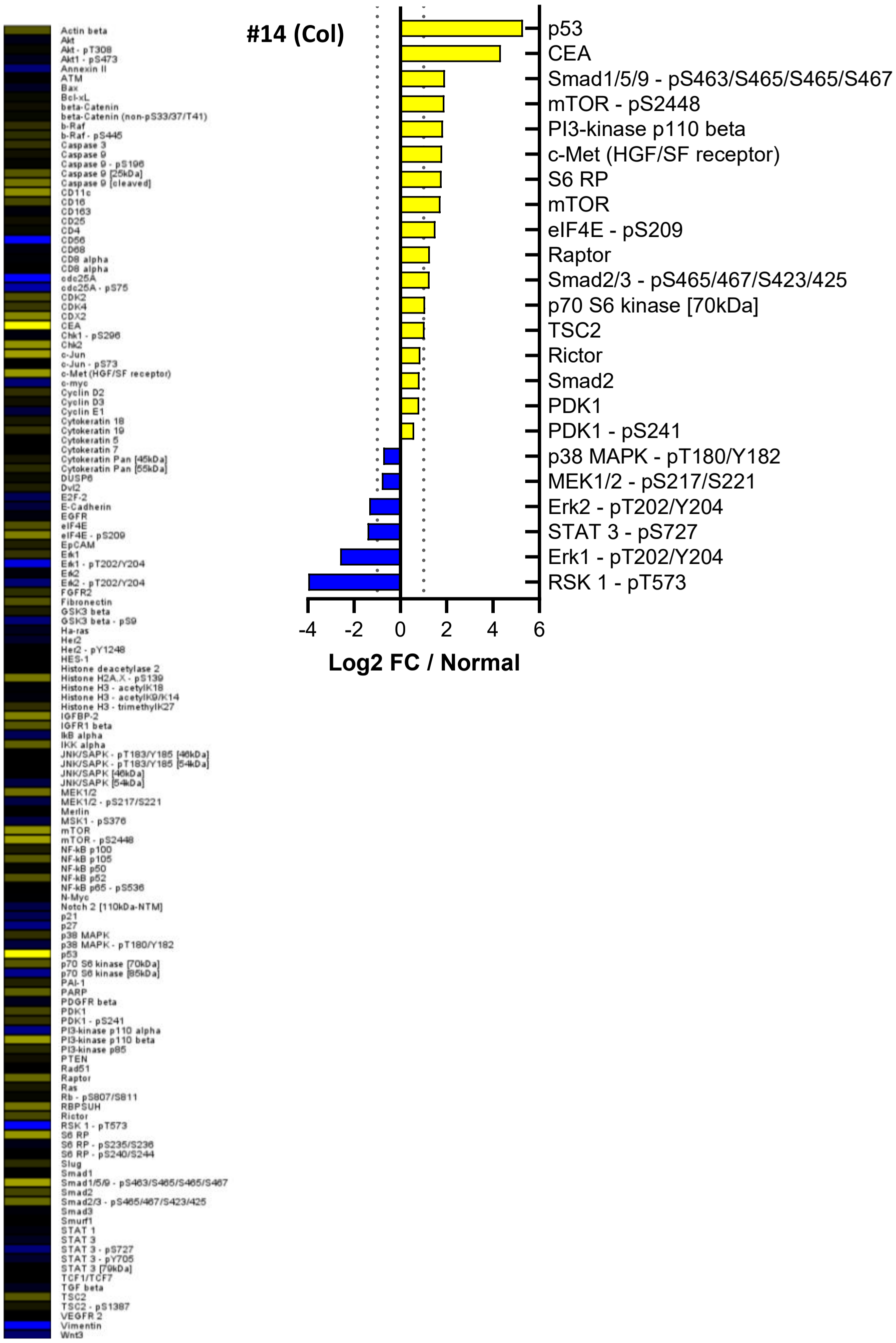

Suppl. Fig. S17: Individual protein profile of colon tumor #14. Heatmap showing tumor/normal relative DigiWest data (Log2 FCs) of all analytes (left) and election of key up- or downregulated analytes versus matched normal tissue (right). Selection was based on markers and key regulatory (pathway) proteins.

Suppl. Fig. S18

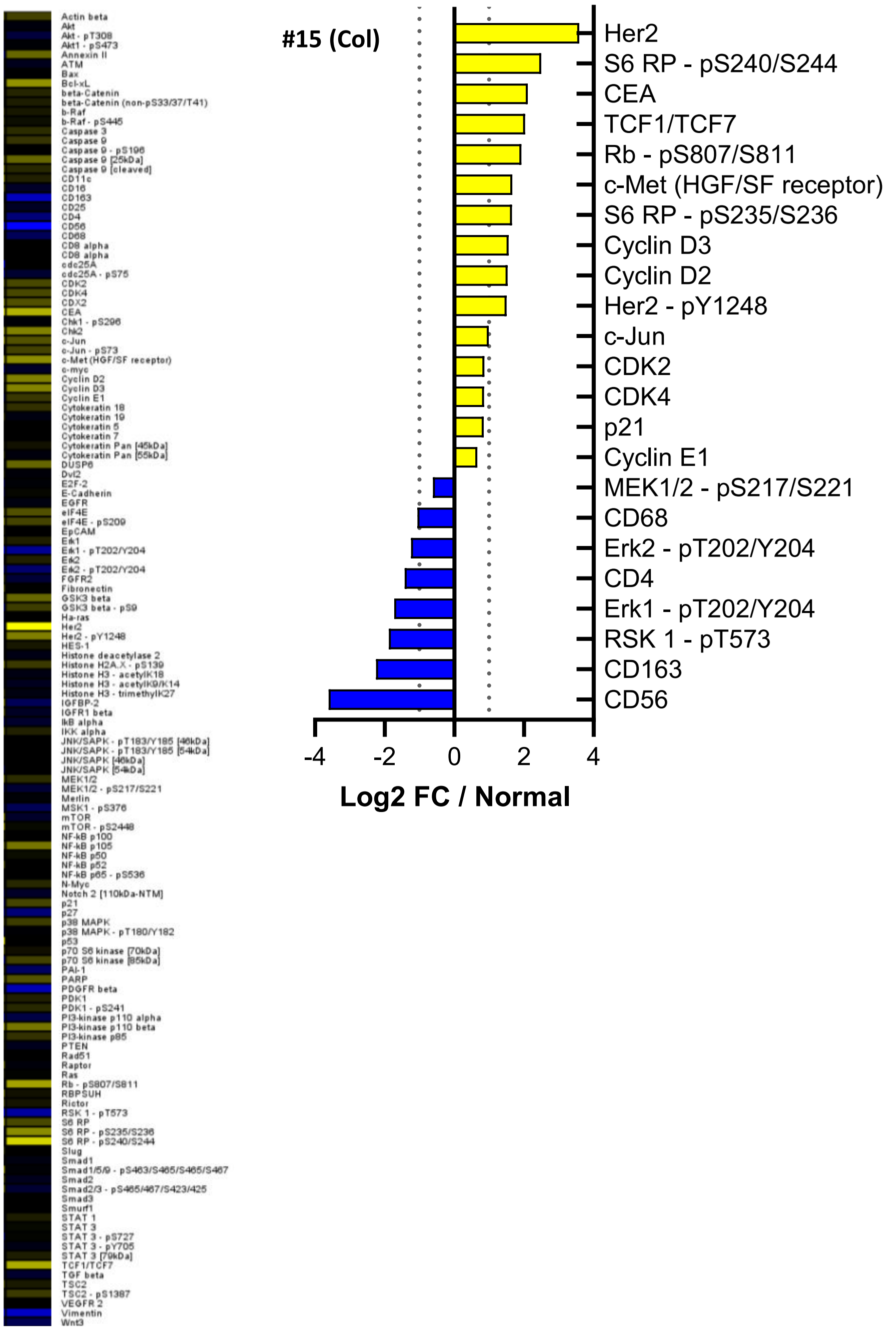

Suppl. Fig. S18: Individual protein profile of colon tumor #15. Heatmap showing tumor/normal relative DigiWest data (Log2 FCs) of all analytes (left) and election of key up- or downregulated analytes versus matched normal tissue (right). Selection was based on markers and key regulatory (pathway) proteins.

Suppl. Fig. S19

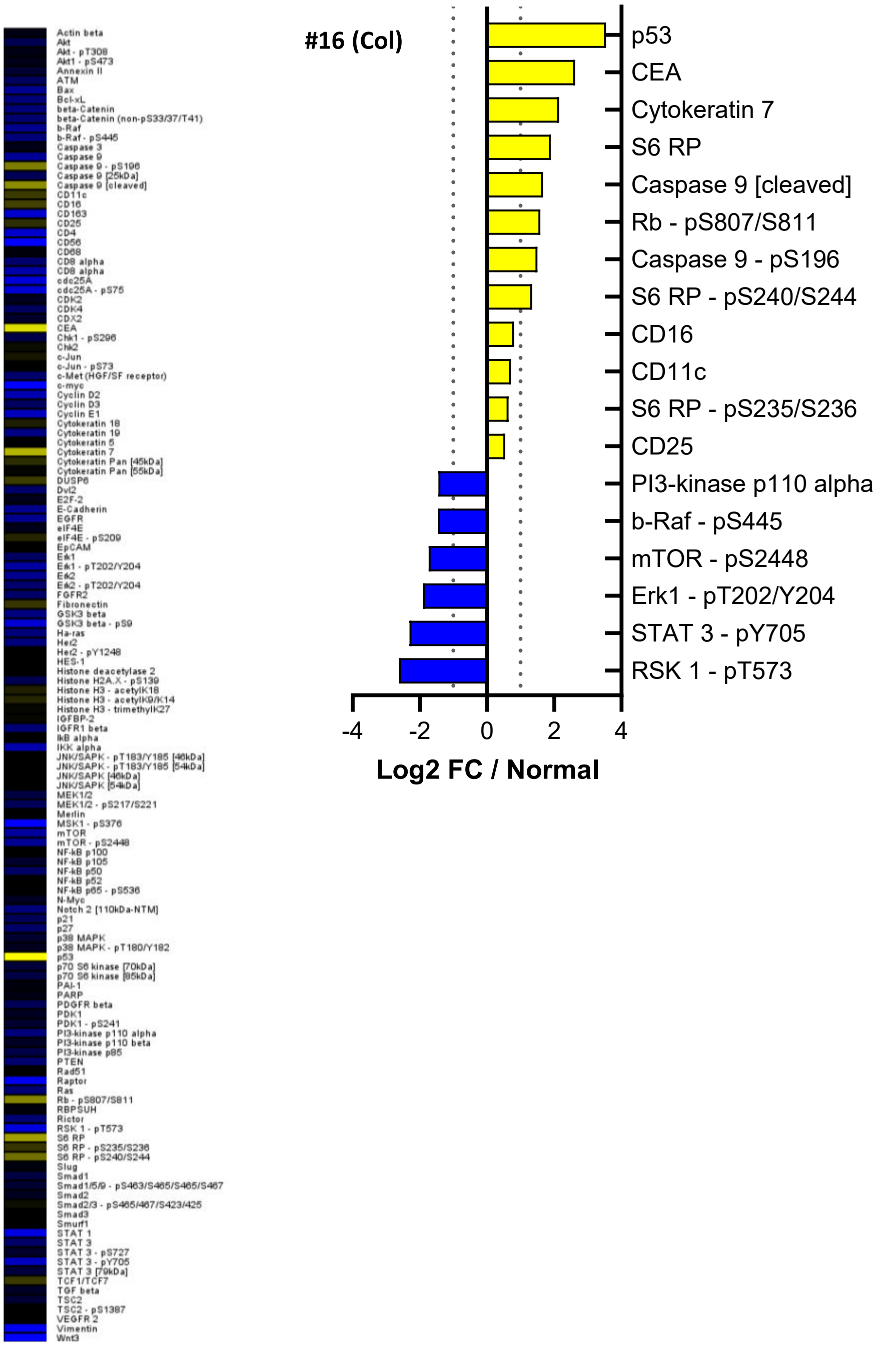

Suppl. Fig. S19: Individual protein profile of colon tumor #16 (**MSI-high tumor**). Heatmap showing tumor/normal relative DigiWest data (Log2 FCs) of all analytes (left) and election of key up- or downregulated analytes versus matched normal tissue (right). Selection was based on markers and key regulatory (pathway) proteins.

Suppl. Fig. S20

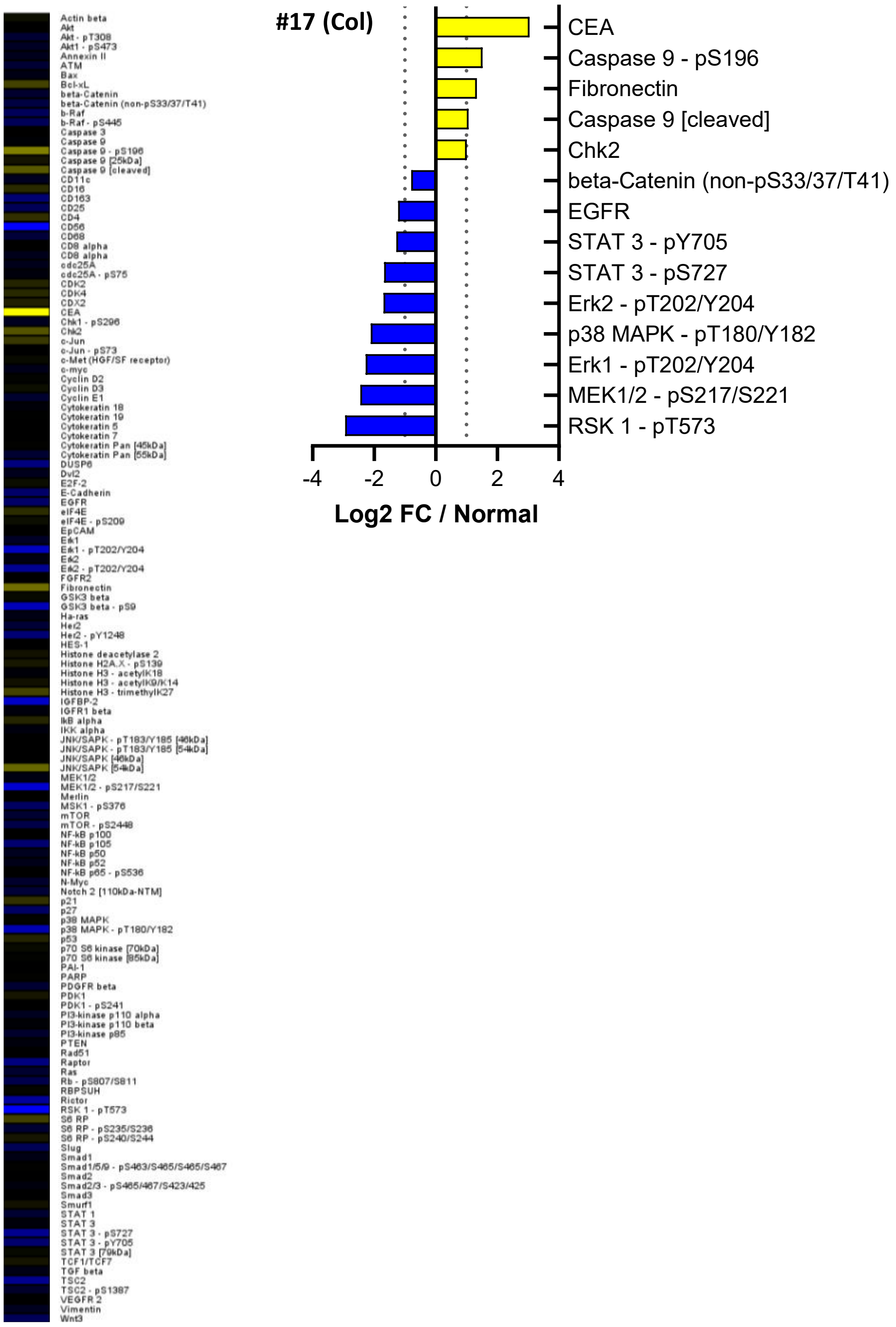

Suppl. Fig. S20: Individual protein profile of colon tumor #17. Heatmap showing tumor/normal relative DigiWest data (Log2 FCs) of all analytes (left) and election of key up- or downregulated analytes versus matched normal tissue (right). Selection was based on markers and key regulatory (pathway) proteins.

Suppl. Fig. S21

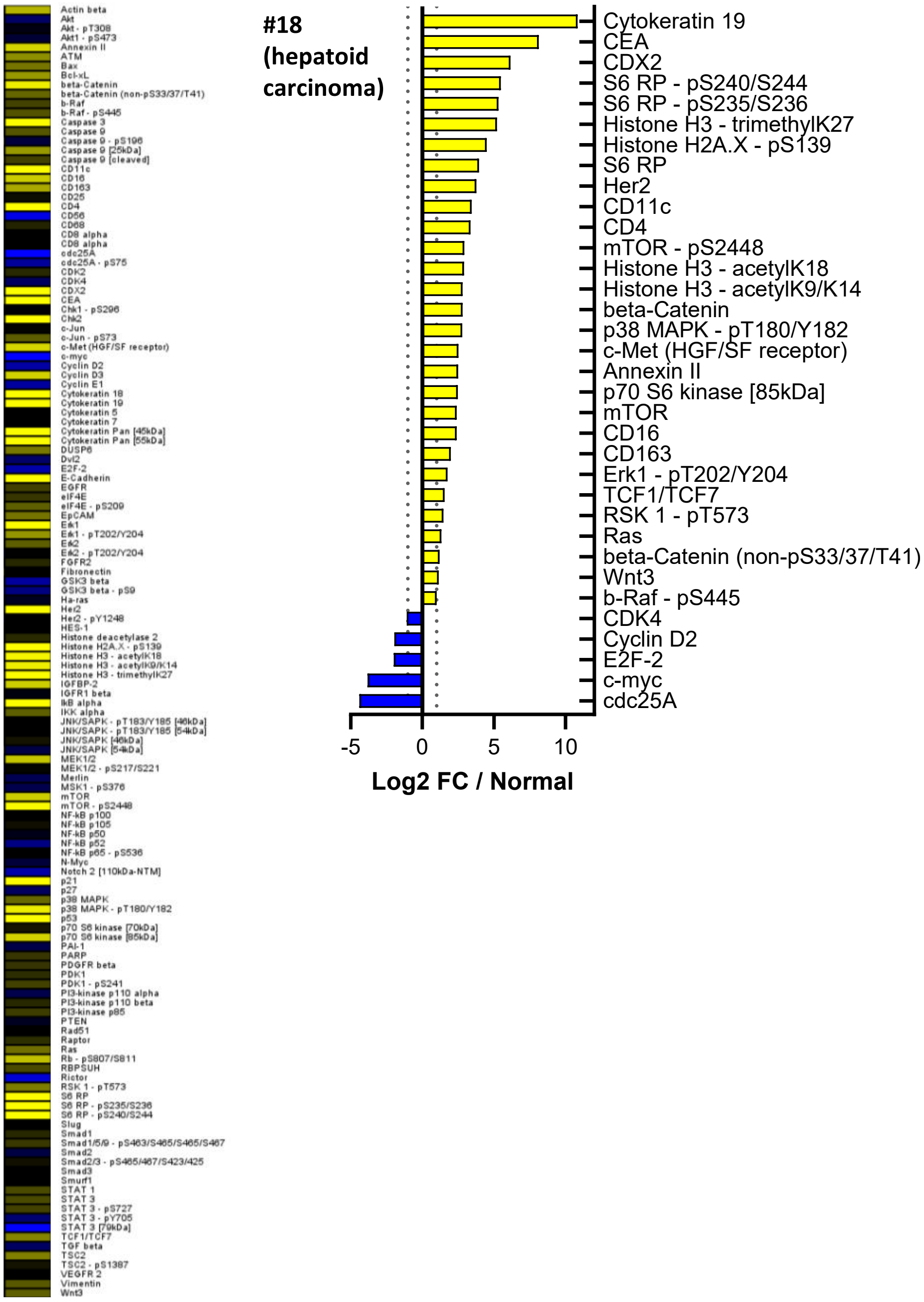

Suppl. Fig. S21: Individual protein profile of colon tumor #18 (**hepatoid carcinoma**). Heatmap showing tumor/normal relative DigiWest data (Log2 FCs) of all analytes (left) and election of key up- or downregulated analytes versus matched normal tissue (right). Selection was based on markers and key regulatory (pathway) proteins.

Suppl. Fig. S22

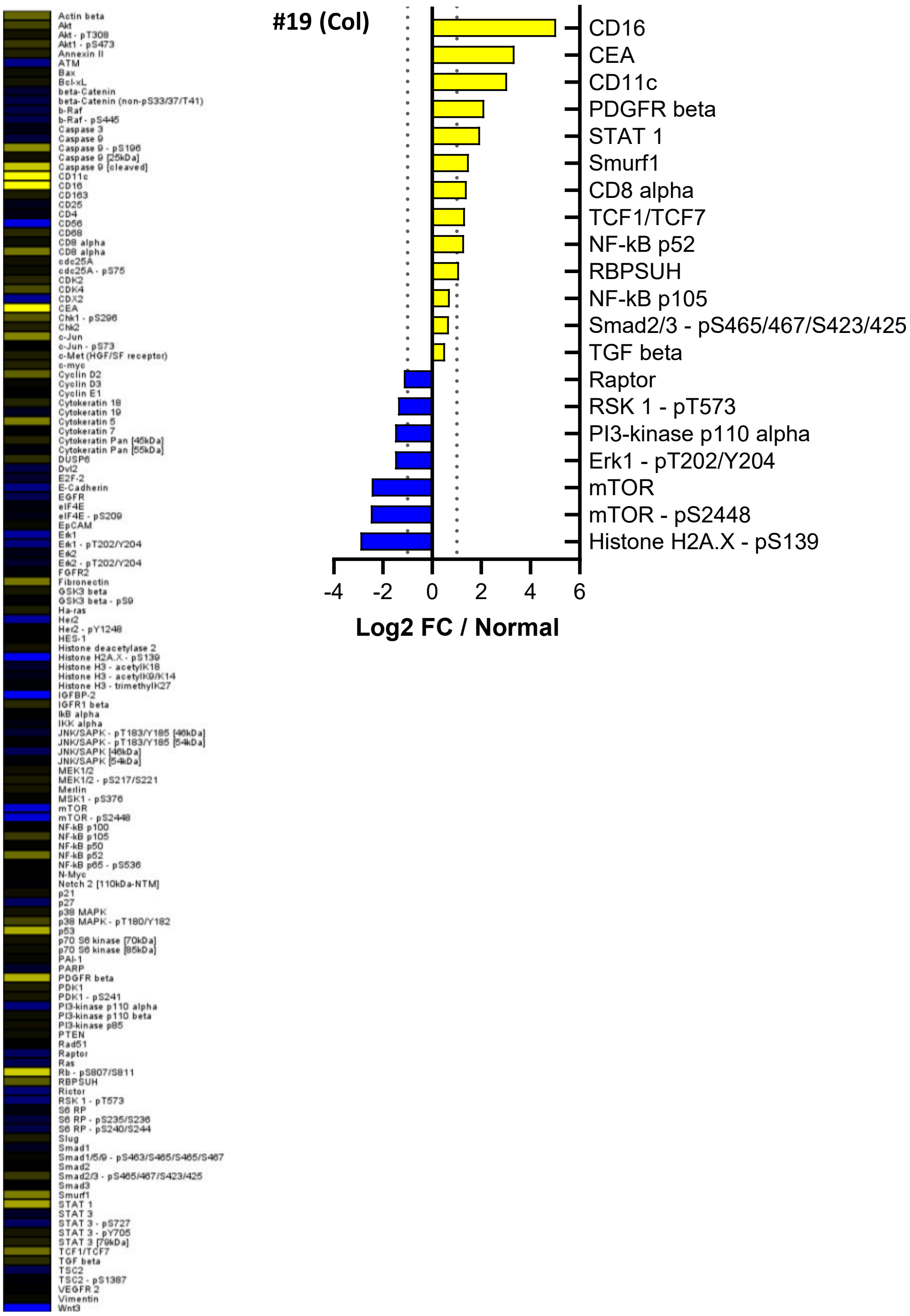

Suppl. Fig. S22: Individual protein profile of colon tumor #19. Heatmap showing tumor/normal relative DigiWest data (Log2 FCs) of all analytes (left) and election of key up- or downregulated analytes versus matched normal tissue (right). Selection was based on markers and key regulatory (pathway) proteins.

Suppl. Fig. S23

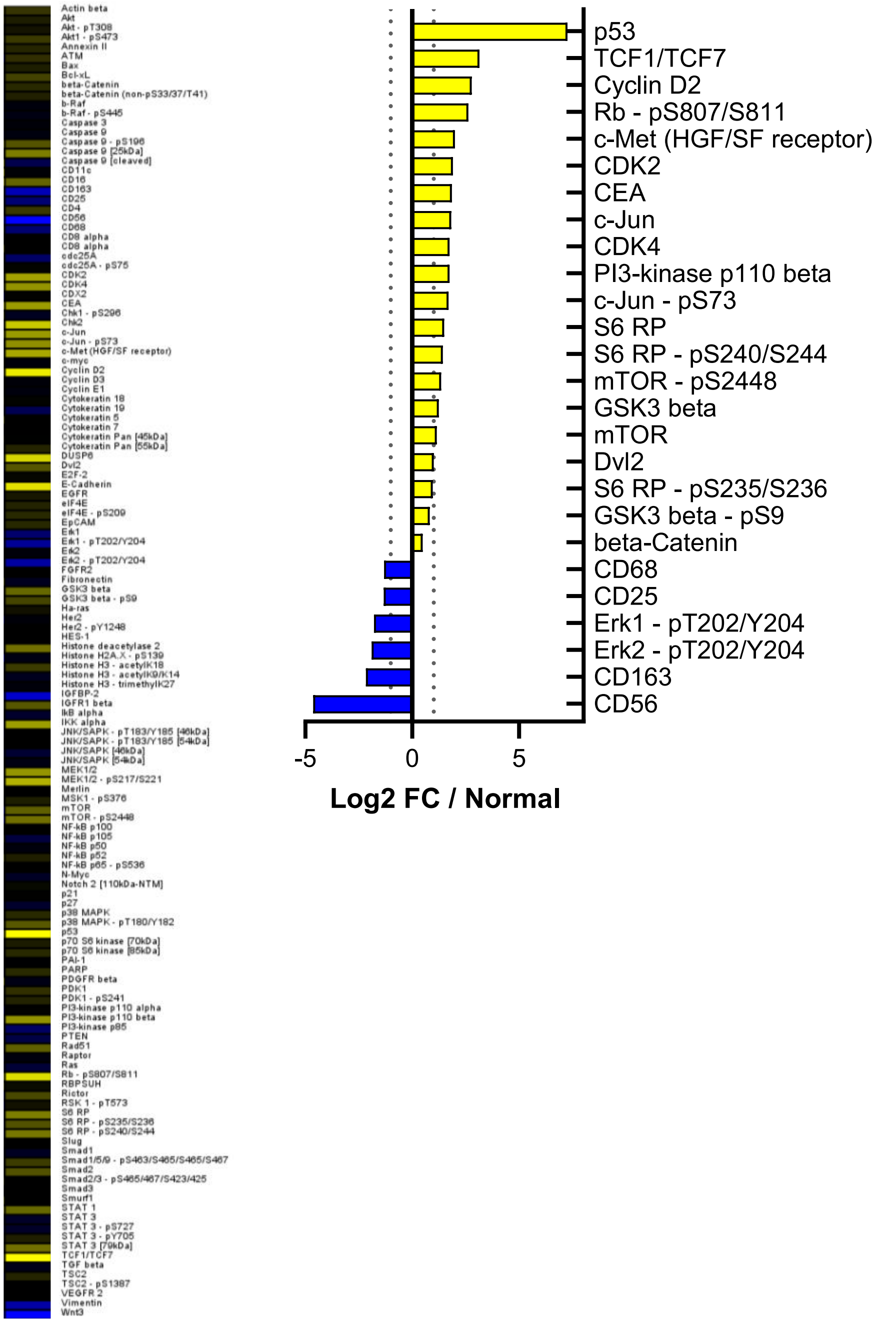

Suppl. Fig. S23: Individual protein profile of colon tumor #19. Heatmap showing tumor/normal relative DigiWest data (Log2 FCs) of all analytes (left) and election of key up- or downregulated analytes versus matched normal tissue (right). Selection was based on markers and key regulatory (pathway) proteins.

Suppl. Fig. S24

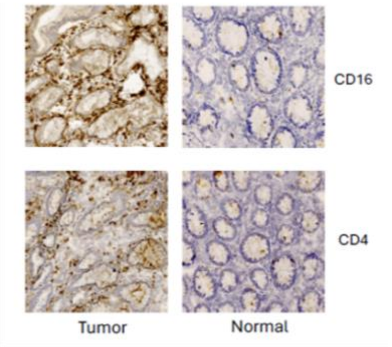

Suppl. Fig. S24: Exemplary immunohistochemical staining of immune-infiltrated tumor #12 (Col) with immune-cell markers CD16 and CD4.

Suppl. Fig. S25

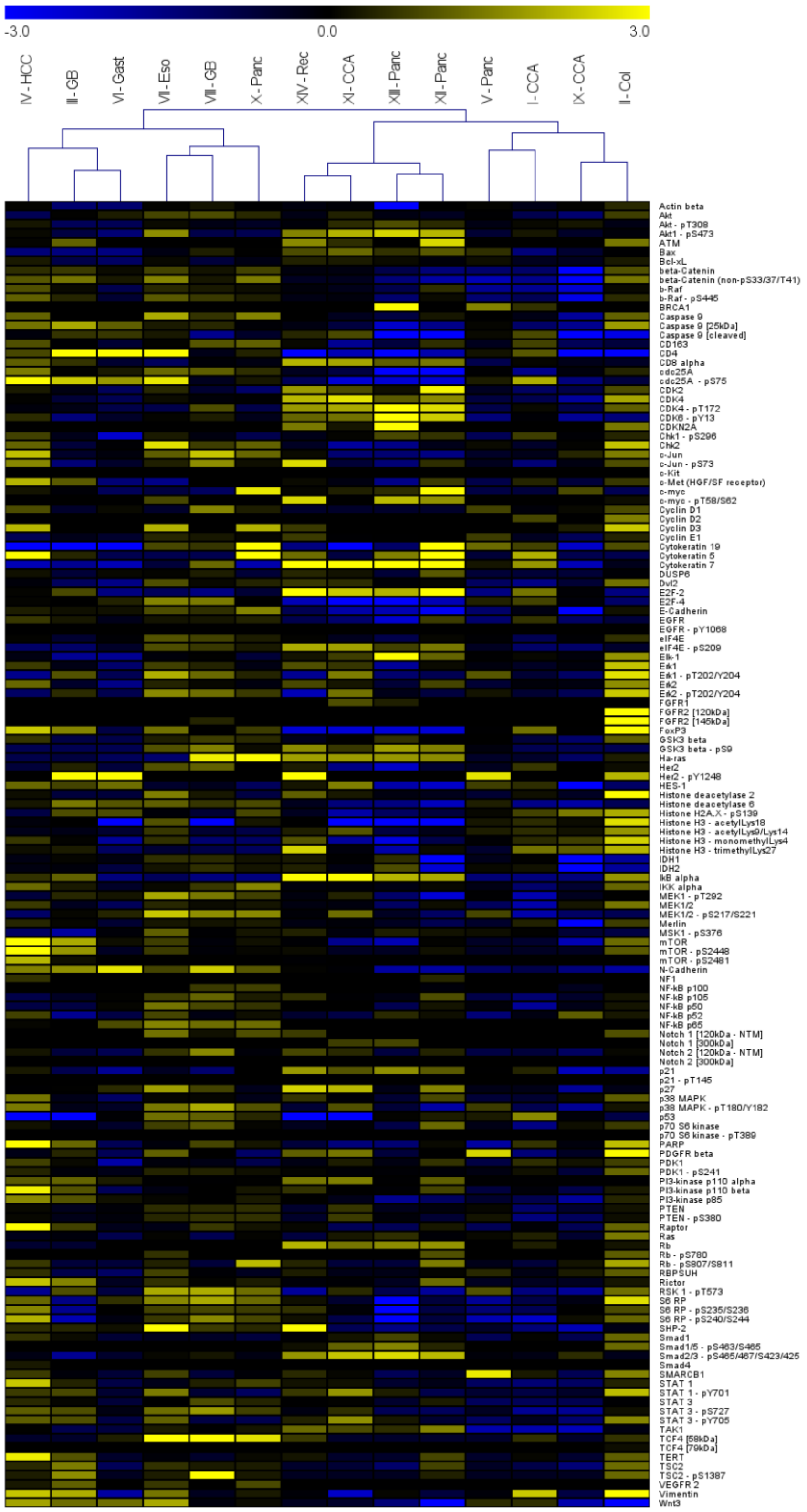

Suppl. Fig. S25: Heatmap and Hierarchical Cluster analysis of DigiWest data set including all non-retrospectively analyzed needle biopsy samples (n = 14). Clustering was performed using Euclidian Distance and complete linkage. HCC = hepatocellular carcinoma, GB = gallbladder carcinoma, Gast = Gastric carcinoma, Eso = esophageal carcinoma, Panc = pancreatic carcinoma, Rec = rectal carcinoma, CCA = cholangiocarcinoma, Col = colon carcinoma.

Suppl. Fig. S26

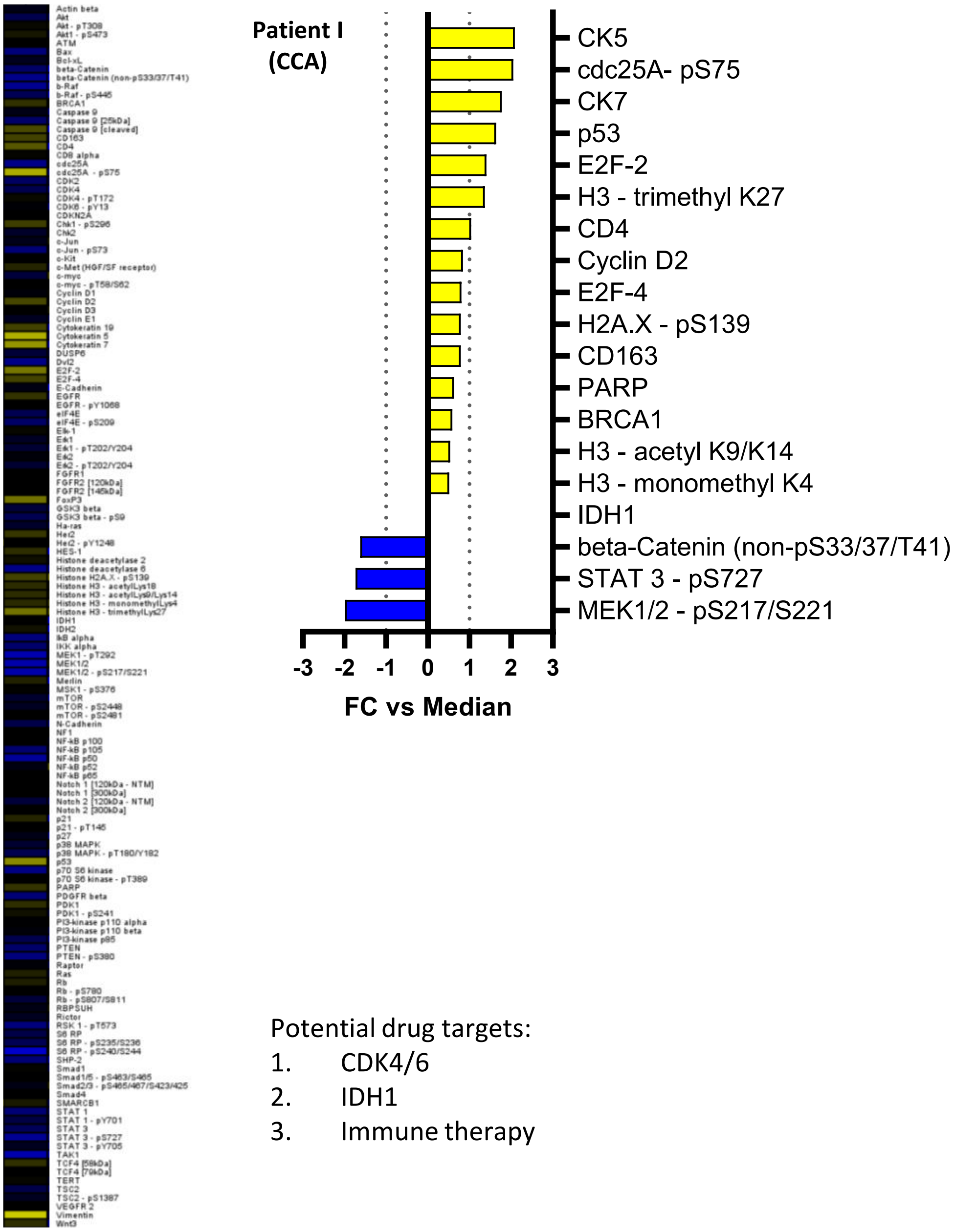

Suppl. Fig. S26: Individual protein profile of patient I (CCA - cholangiocarcinoma). Left: Heatmap of DigiWest expression data (normalized AFI) as Log2 FC in relation to median signal (baseline) across all tumors (I-XIV). Right: Selection of key analytes shown relative to baseline signal. Bottom: Ranking of potential drug targets based on DigiWest data.

Suppl. Fig. S27

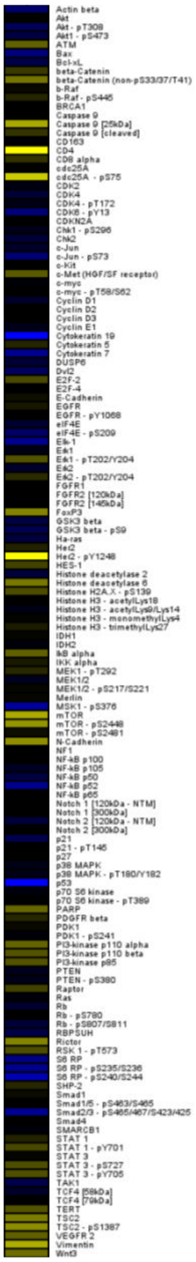

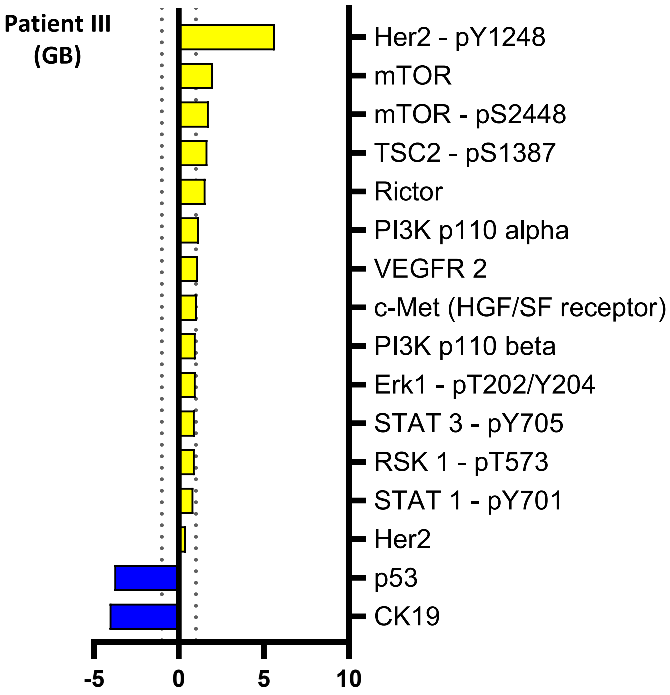

Potential drug targets:

1. mTOR
2. Her2
3. TKI (Her2/VEGFR/MET)
4. MEK

Suppl. Fig. S27: Individual protein profile of patient III (GB – gallbladder carcinoma). Left: Heatmap of DigiWest expression data (normalized AFI) as Log2 FC in relation to median signal (baseline) across all tumors (I-XIV). Right: Selection of key analytes shown relative to baseline signal. Bottom: Ranking of potential drug targets based on DigiWest data.

Suppl. Fig. S28

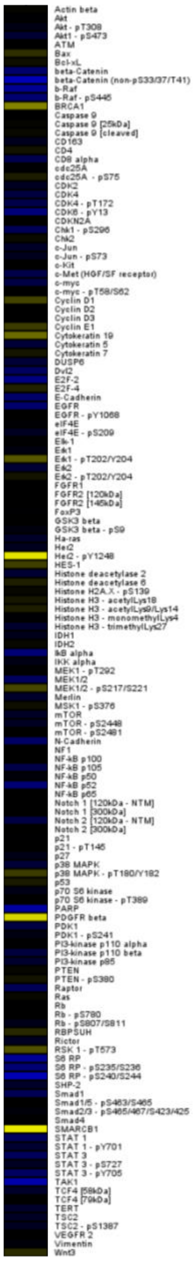

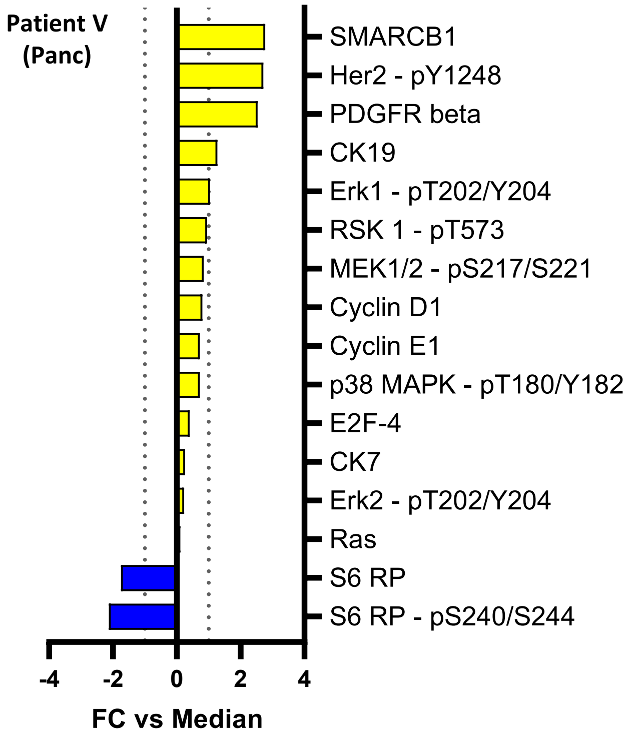

Potential drug targets:

1. KRAS
2. PDGFR
3. Her2
4. CDK4/6

Suppl. Fig. S28: Individual protein profile of patient V (Panc – pancreatic carcinoma). Left: Heatmap of DigiWest expression data (normalized AFI) as Log2 FC in relation to median signal (baseline) across all tumors (I-XIV). Right: Selection of key analytes shown relative to baseline signal. Bottom: Ranking of potential drug targets based on DigiWest data.

Suppl. Fig. S29

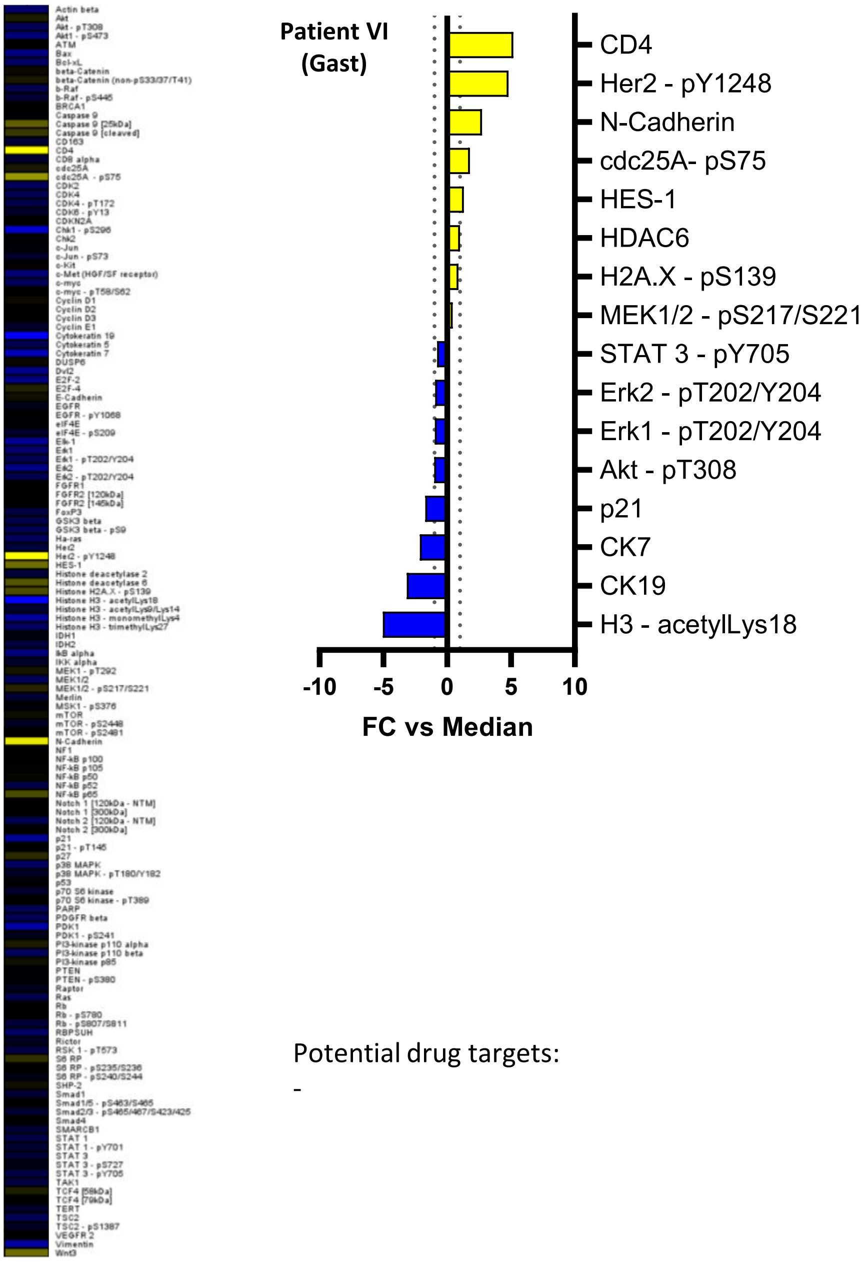

Suppl. Fig. S29: Individual protein profile of patient VI (Gast – gastric carcinoma). Left: Heatmap of DigiWest expression data (normalized AFI) as Log2 FC in relation to median signal (baseline) across all tumors (I-XIV). Right: Selection of key analytes shown relative to baseline signal. Bottom: Ranking of potential drug targets based on DigiWest data.

Suppl. Fig. S30

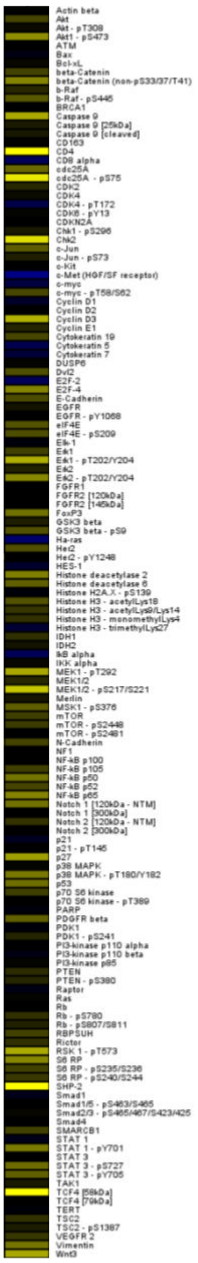

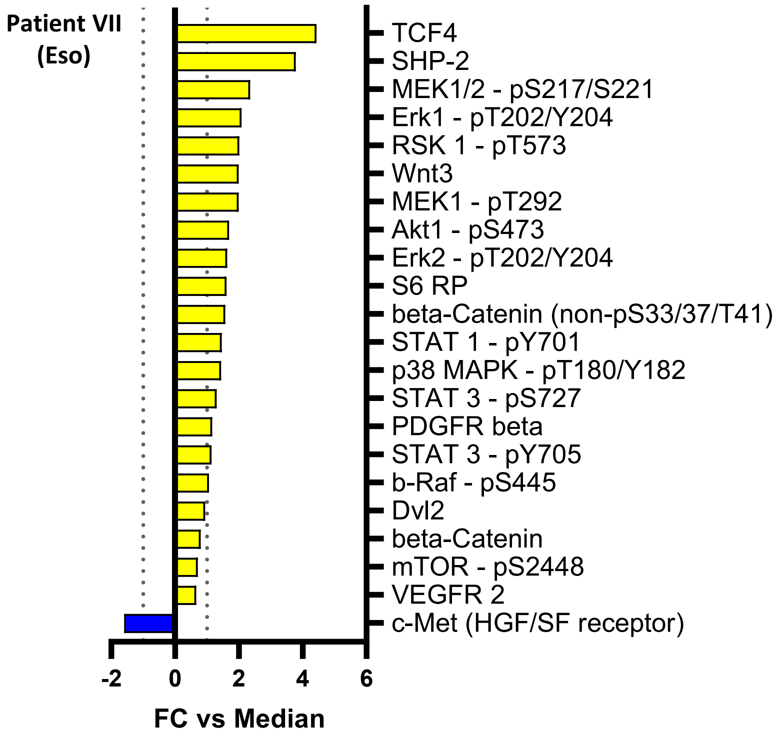

Potential drug targets:

1. FGFR
2. TK (PDGFR/VEGFR)

Suppl. Fig. S30: Individual protein profile of patient VII (Eso – esophageal carcinoma). Left: Heatmap of DigiWest expression data (normalized AFI) as Log2 FC in relation to median signal (baseline) across all tumors (I-XIV). Right: Selection of key analytes shown relative to baseline signal. Bottom: Ranking of potential drug targets based on DigiWest data. Eventual MTB treatment printed in bold.

Suppl. Fig. S31

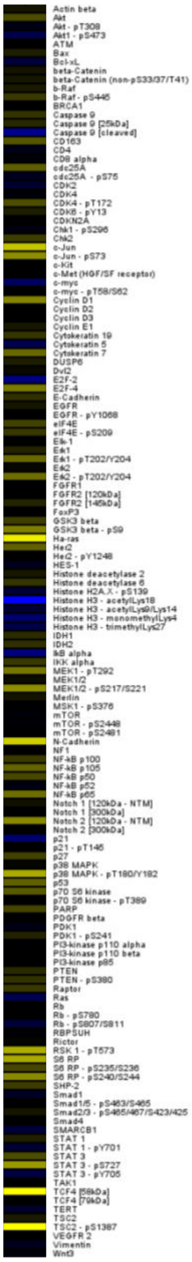

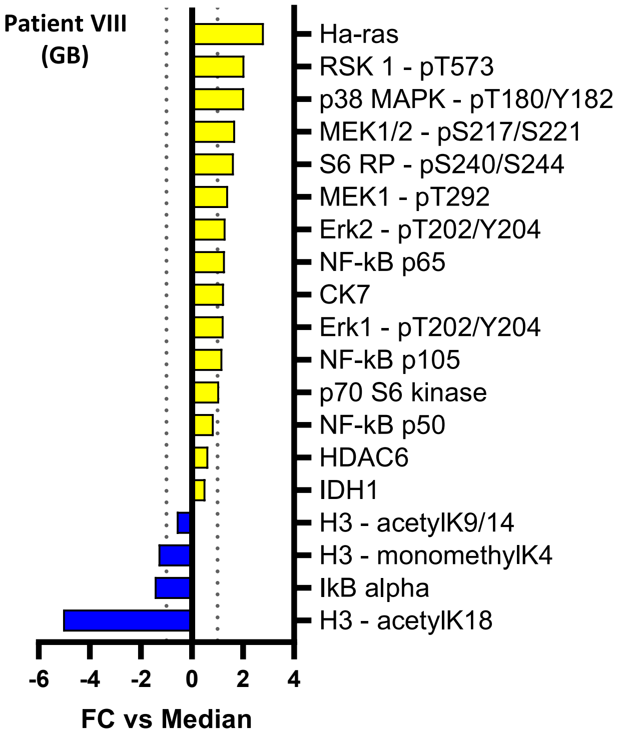

Potential drug targets:

1. MEK
2. HDAC

Suppl. Fig. S31: Individual protein profile of patient VIII (GB – gallbladder carcinoma). Left: Heatmap of DigiWest expression data (normalized AFI) as Log2 FC in relation to median signal (baseline) across all tumors (I-XIV). Right: Selection of key analytes shown relative to baseline signal. Bottom: Ranking of potential drug targets based on DigiWest data.

Suppl. Fig. S32

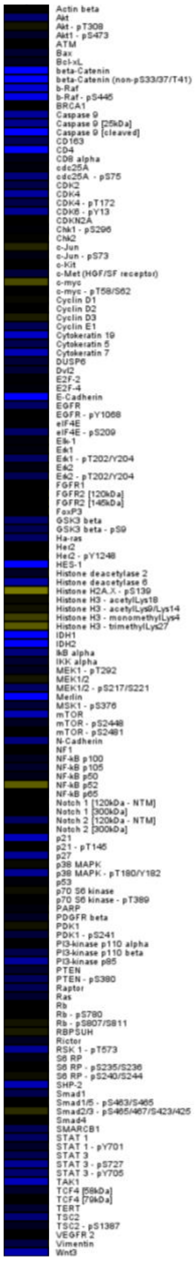

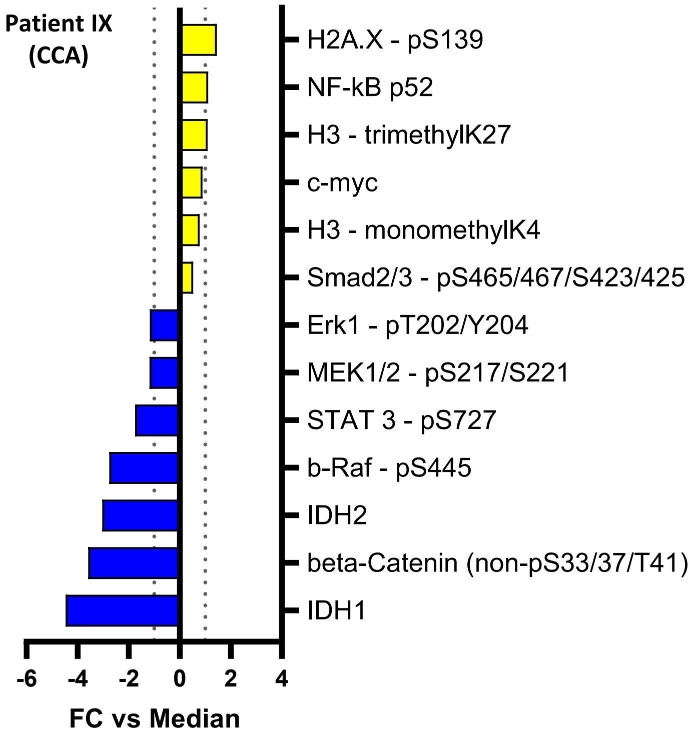

Potential drug targets:

-

Suppl. Fig. S32: Individual protein profile of patient IX (CCA – cholangiocarcinoma). Left: Heatmap of DigiWest expression data (normalized AFI) as Log2 FC in relation to median signal (baseline) across all tumors (I-XIV). Right: Selection of key analytes shown relative to baseline signal. Bottom: Ranking of potential drug targets based on DigiWest data.

Suppl. Fig. S33

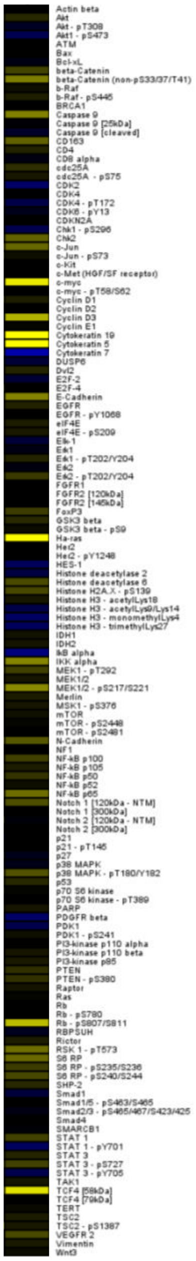

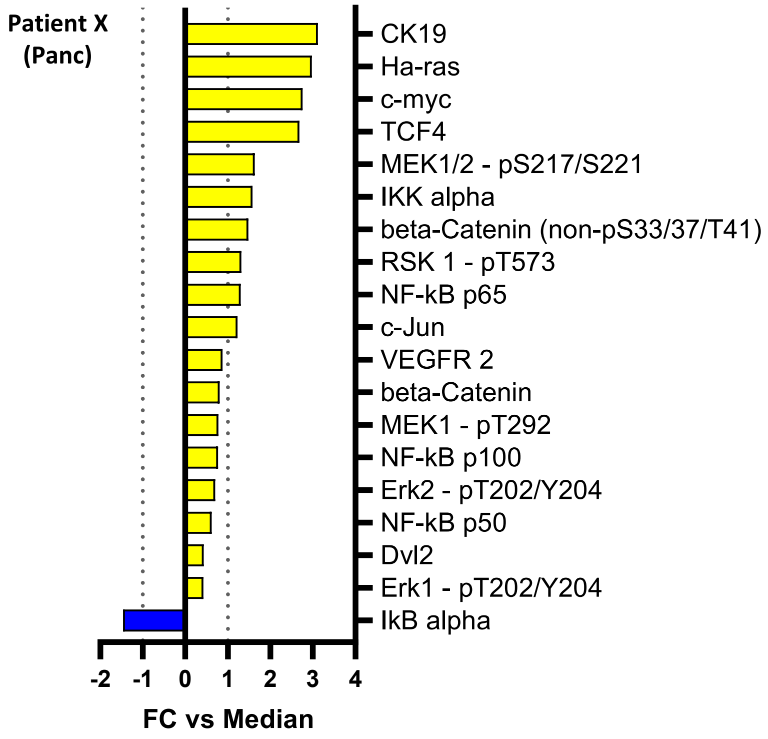

Potential drug targets:

1. MEK
2. KRAS
3. VEGFR

Suppl. Fig. S33: Individual protein profile of patient X (Panc – pancreatic carcinoma). Left: Heatmap of DigiWest expression data (normalized AFI) as Log2 FC in relation to median signal (baseline) across all tumors (I-XIV). Right: Selection of key analytes shown relative to baseline signal. Bottom: Ranking of potential drug targets based on DigiWest data.

Suppl. Fig. S34

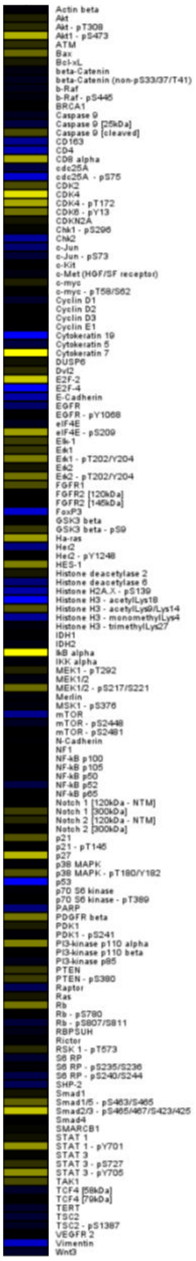

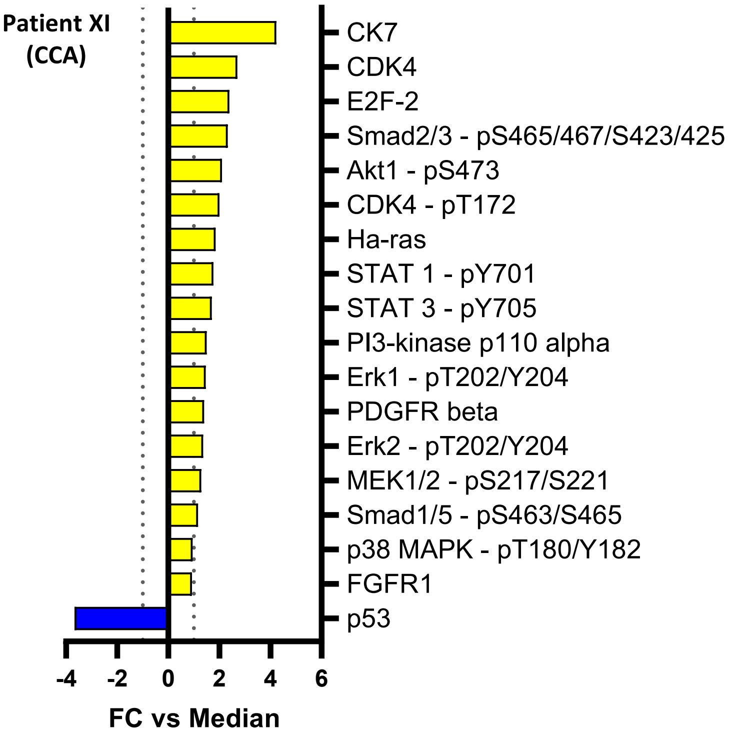

Potential drug targets:

1. TK (PDGFR/FGFR)
2. CDK4/6
3. TGFBR

Suppl. Fig. S34: Individual protein profile of patient XI (CCA – cholangiocarcinoma). Left: Heatmap of DigiWest expression data (normalized AFI) as Log2 FC in relation to median signal (baseline) across all tumors (I-XIV). Right: Selection of key analytes shown relative to baseline signal. Bottom: Ranking of potential drug targets based on DigiWest data.

Suppl. Fig. S35

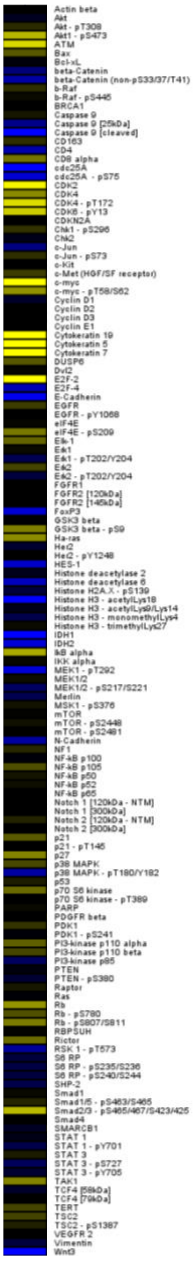

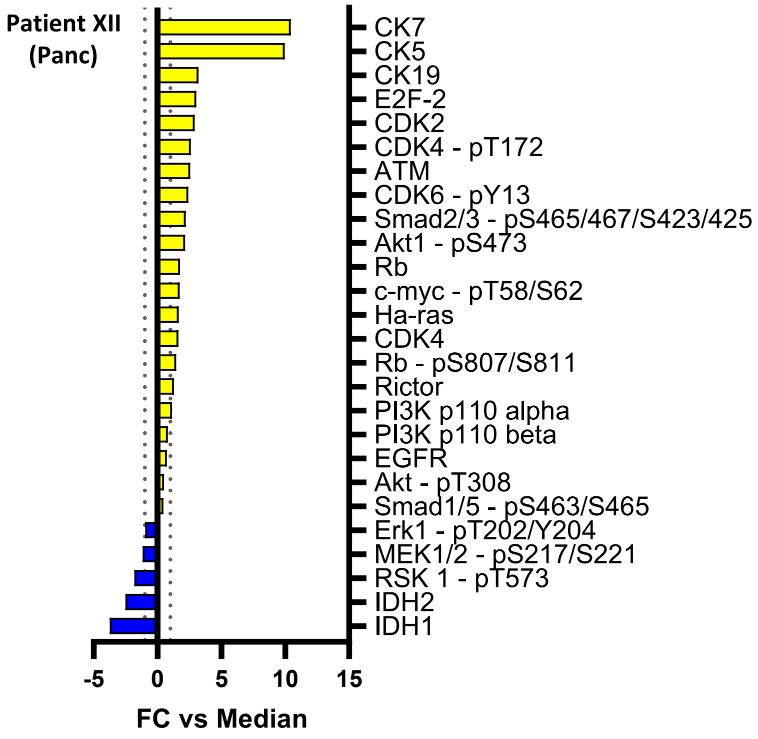

Potential drug targets:

1. CDK4/6
2. PI3K
3. mTOR

Suppl. Fig. S35: Individual protein profile of patient XII (Panc – pancreatic carcinoma). Left: Heatmap of DigiWest expression data (normalized AFI) as Log2 FC in relation to median signal (baseline) across all tumors (I-XIV). Right: Selection of key analytes shown relative to baseline signal. Bottom: Ranking of potential drug targets based on DigiWest data.

Suppl. Fig. S36

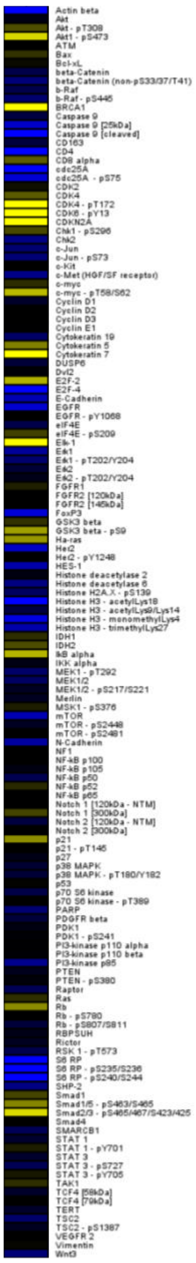

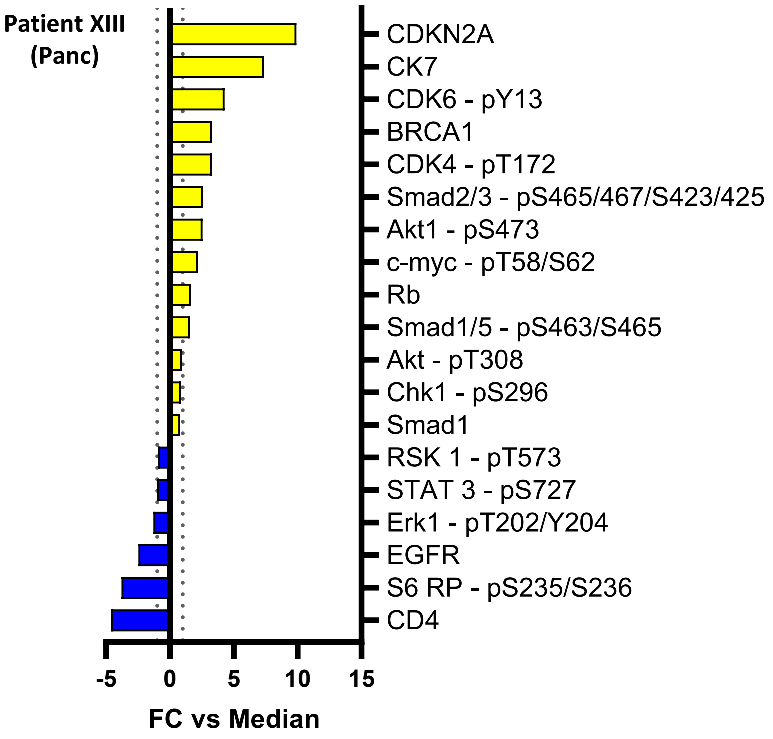

Potential drug targets:

1. CDK4/6
2. TGFBR

Suppl. Fig. S36: Individual protein profile of patient XIII (Panc – pancreatic carcinoma). Left: Heatmap of DigiWest expression data (normalized AFI) as Log2 FC in relation to median signal (baseline) across all tumors (I-XIV). Right: Selection of key analytes shown relative to baseline signal. Bottom: Ranking of potential drug targets based on DigiWest data.

Suppl. Fig. S37

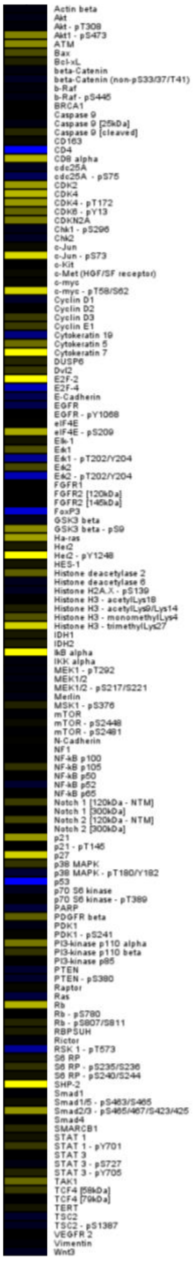

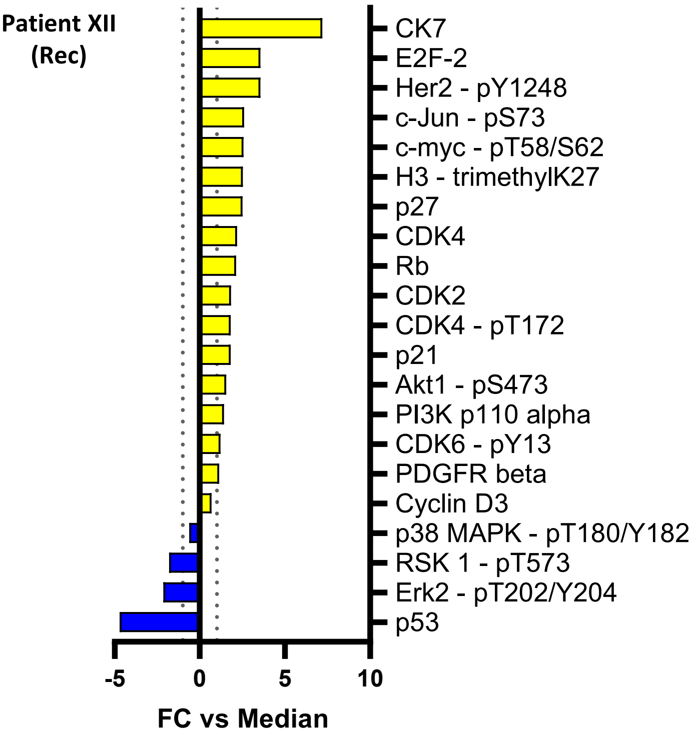

Potential drug targets:

1. CDK4/6
2. Her2
3. PI3K

Suppl. Fig. S37: Individual protein profile of patient XIV (Rec – rectal carcinoma). Left: Heatmap of DigiWest expression data (normalized AFI) as Log2 FC in relation to median signal (baseline) across all tumors (I-XIV). Right: Selection of key analytes shown relative to baseline signal. Bottom: Ranking of potential drug targets based on DigiWest data.

Suppl. Fig. S38

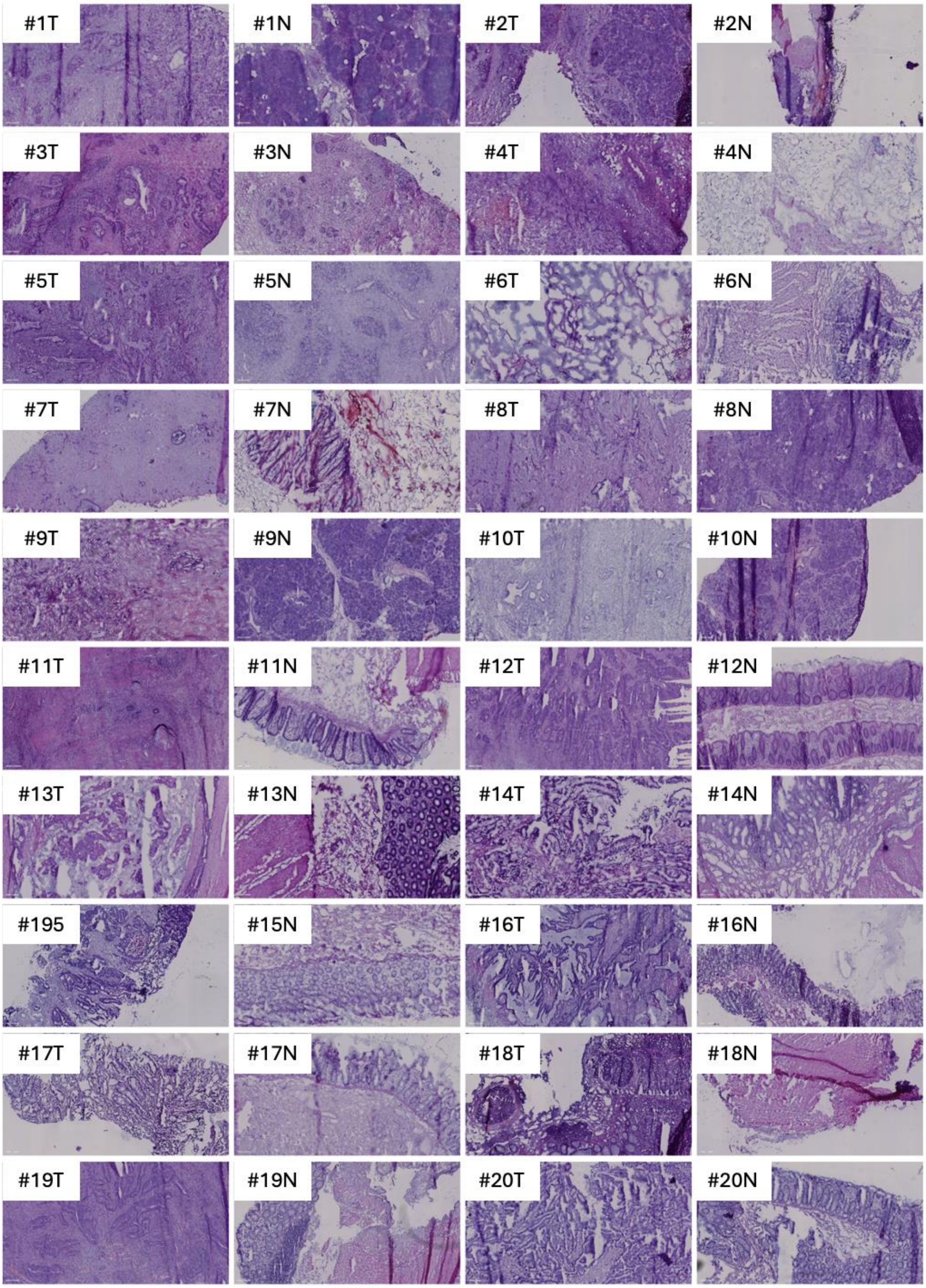

Suppl. Fig. S38: Hematoxylin and Eosin (H&E) staining images of retrospectively analyzed tumor and normal tissue sections from all samples from the retrospective cohort (#1 - #20; also see Table 1). Tumor/normal tissue image pairs are indicated as T = Tumor, N = Normal. Comments when applicable: **#1T**: 40% tumor, 60% stroma. **#2T**: 5% tumor, 95% normal tissue. **#3T**: 60% tumor, 40% stroma. **#3N**: 70% fat, 20% stroma, 10% parenchyma. **#4T**: 30% tumor, 70% stroma. **#4N**: fat/connective tissue only, no parenchyma. **#5T**: 20% tumor, 75% stroma, 5% parenchyma. **#6T**: 1% tumor, rest mucin/stroma. **#6N**: 5% parenchyma, 95% connective/muscle tissue. **#7T**: 5% tumor, 90% stroma, 5% normal tissue. **#7N**: fat/lymph tissue only, no parenchyma. **#8T**: 35% tumor, 60% stroma, 5% normal tissue. **#9T**: 10% tumor, 90% stroma. **#10T**: <1% tumor, ~95% stroma, 5% parenchyma. **#11T**: <1% tumor, ~90% stroma/connective/muscle tissue, 10% normal mucosa. **#12T**: 75% tumor, 25% stroma. **#13T**: 20% tumor, 75% muscle, 5% mucosa. **#14T**: 90% tumor, 10% stroma. **#15T**: 70% tumor (invasive), 30% stroma. **#16T**: 80% tumor/mucin, 20% stroma. **#16N**: poor tissue section. **#17T**: 50% tumor, 50% stroma. **#18T**: 10% tumor, 50% stroma/muscle tissue, 40% normal mucosa. **#18N**: muscle only, no mucosa. **#19T**: 50% tumor, 50% stroma. **#20T**: 80% tumor, 20% stroma.

II. Supplementary Tables

Suppl. Table 1

Marker (Analyte)	Gene	Associated Immune Cell Type(s)
**CD16**	FCGR3A	NK (Natural Killer) Cells, Neutrophils
**CD163**	CD163	M2 Macrophages
**CD4**	CD4	T Cells
**CD68**	CD68	Macrophages
**CD8 alpha**	CD8A	Cytotoxic T Cells
**CD25**	IL2RA	Regulatory T Cells (TRegs)
**CD11c**	ITGAX	Dendritic Cells
**CD56**	NCAM1	NK (Natural Killer) Cells

Suppl. Table 1: Immune cell markers employed in retrospective DigiWest analysis and their respective associated immune cell types.

Supplementary Data 1

All primary antibodies used for DigiWest in retrospective and prospective study (enclosed Excel file).

Supplementary Data 2

All raw and normalized DigiWest data from the retrospective part of the study (enclosed Excel file).

Supplementary Data 3

All raw and normalized DigiWest data from the prospective part of the study (enclosed Excel file).
